# Longitudinal Sodium Dynamics After Aneurysmal Subarachnoid Hemorrhage: A Single-Center Cohort Study with a Contemporary Literature Review

**DOI:** 10.3390/biomedicines14092081

**Published:** 2026-09-16

**Authors:** Adrianna Lebiedzińska, Jarosław Kędziora, Jowita Woźniak, Katarzyna Smarzewska, Waldemar Goździk, Małgorzata Burzyńska

**Affiliations:** 1 Clinical Department of Anesthesiology and Intensive Therapy, University Clinical Hospital in Wroclaw, 50-556 Wroclaw, Poland; 2 Clinical Department of Anesthesiology and Intensive Therapy, Faculty of Medicine, Wroclaw Medical University, 50-367 Wroclaw, Poland; 3 Clinical Department of Neurosurgery, University Centre for Neurology and Neurosurgery, Wroclaw Medical University, 50-367 Wroclaw, Poland; 4 Clinical Department of Neurosurgery, Faculty of Medicine, Wroclaw Medical University, 50-367 Wroclaw, Poland

**Keywords:** aneurysmal subarachnoid hemorrhage, sodium variability, interday variability, hypernatremia, hyponatremia, delayed cerebral ischemia, delayed neurological deficit, neurocritical care, SEBES

## Abstract

**Background and Objectives:** Disorders of sodium homeostasis are common after aneurysmal subarachnoid hemorrhage (aSAH), but the prognostic value of longitudinal sodium dynamics remains unclear. We evaluated whether dynamic sodium parameters provide prognostic information beyond admission sodium concentration. **Methods:** This retrospective cohort study included 232 consecutive patients with angiographically confirmed aSAH admitted between January 2014 and December 2025. Serial serum sodium measurements were used to assess admission sodium, mean sodium, interday sodium variability, and hypernatremia burden. Associations with clinical outcomes were evaluated using multivariable regression, survival analysis, receiver operating characteristic analysis, and linear mixed-effects models. A landmark analysis was performed to assess the prognostic value of sodium variability during the first 5 days after admission. **Results:** Admission sodium concentration showed limited discrimination for 30-day mortality (area under the curve [AUC] 0.606, 95% confidence interval [CI] 0.509–0.703). Greater interday sodium variability was independently associated with mortality (odds ratio [OR] 1.55, 95% CI 1.29–1.86; *p* < 0.001) and remained significant after adjustment for severe hypernatremia (OR 1.32, 95% CI 1.09–1.60; *p* = 0.005). Adding interday variability to the baseline clinical model improved its discriminative performance (AUC 0.845 vs. 0.899; ΔAUC 0.054, 95% CI 0.010–0.099; *p* = 0.017). The association remained significant in the adjusted landmark analysis (hazard ratio [HR] 1.21, 95% CI 1.13–1.29; *p* < 0.001). Greater sodium variability was also associated with poorer neurological outcomes. **Conclusions:** Longitudinal sodium dynamics provide prognostic information beyond admission sodium concentration in patients with aSAH. Greater interday sodium variability was independently associated with 30-day mortality and remained robust after accounting for potential immortal-time bias. Prospective multicenter studies are warranted to validate its prognostic value and the cohort-derived variability threshold.

## 1. Introduction

Aneurysmal subarachnoid hemorrhage (aSAH) is a devastating cerebrovascular disorder associated with high mortality and long-term neurological disability, despite major advances in aneurysm treatment and neurocritical care [1,2]. Secondary neurological and systemic complications remain key determinants of patient outcome [1,2].

Disorders of sodium homeostasis are among the most frequent metabolic complications following aSAH [3,4]. They may result from acute brain injury, hypothalamic–pituitary dysfunction, or treatment-related factors [5,6]. Hyponatremia affects approximately 30–50% of patients and is most commonly attributed to the syndrome of inappropriate antidiuretic hormone secretion (SIADH) or cerebral salt wasting (CSW), two conditions with overlapping biochemical features but distinct pathophysiological mechanisms and therapeutic implications [4,7]. Consequently, earlier studies largely focused on the pathophysiology, differential diagnosis, and management of hyponatremia [7,8,9].

Hypernatremia, although traditionally receiving less attention than hyponatremia, is also clinically relevant after aSAH. It may reflect impaired free-water homeostasis, hypothalamic–pituitary dysfunction, including central diabetes insipidus, severe neurological injury, or treatment-related factors such as hyperosmolar therapy and exogenous sodium administration [5,6,10,11]. Several studies have associated hypernatremia with increased mortality and unfavorable neurological outcomes [10,11,12,13], whereas its relationship with delayed cerebral ischemia (DCI) and angiographic vasospasm has been less consistent [12,14,15,16]. These findings suggest that the entire spectrum of sodium disturbances, rather than hyponatremia alone, should be considered when evaluating sodium homeostasis after aSAH.

More recently, attention has shifted from individual sodium abnormalities toward the dynamic longitudinal behavior of serum sodium throughout the course of hospitalization. Admission sodium concentration appears to have limited prognostic value [14,17,18], whereas longitudinal sodium trajectories—including mean serum sodium concentration, sodium variability, and day-to-day fluctuations—have shown stronger and more consistent associations with disease severity and clinical outcome [14,15,19,20,21]. Greater sodium variability has been independently associated with increased mortality [14,20,21], unfavorable functional outcome [20,21], prolonged ICU stay, and longer hospitalization [15,20].

Whether sodium dysregulation contributes to, or merely reflects, secondary brain injury remains uncertain. In particular, the relationship between sodium dysregulation and DCI remains controversial. Earlier studies suggested that hyponatremia may precede cerebral vasospasm (CV) and therefore serve as an early marker of impending DCI [9]. In contrast, more recent longitudinal studies have shown that, although higher sodium concentrations and greater sodium variability are associated with worse neurological outcomes [14,15,19,20,21], they do not independently predict DCI or angiographic vasospasm after adjustment for hemorrhage severity and other confounding factors [14,16]. Thus, the prognostic significance of sodium dynamics may extend beyond their potential relationship with vasospasm or DCI.

Another unresolved issue is the association between early brain injury (EBI) and subsequent sodium dysregulation. Hypothalamic injury occurring immediately after aneurysm rupture may disrupt sodium and water homeostasis, raising the possibility that markers of EBI could identify patients at increased risk of clinically relevant sodium disturbances. The Subarachnoid Hemorrhage Early Brain Edema Score (SEBES) is a validated imaging marker of EBI severity and has been associated with poor neurological outcome [5,22,23]. However, its association with longitudinal sodium trajectories has not been adequately characterized. Similarly, the influence of aneurysm characteristics, neurological severity, and treatment-related hypernatremia on sodium trajectories has not been fully elucidated.

Therefore, the present study comprehensively evaluated sodium homeostasis in a contemporary cohort of patients with aSAH treated at a tertiary neurocritical care center. We examined several complementary measures of sodium regulation—including admission serum sodium concentration, mean sodium concentration, overall sodium variability, interday sodium variability, and hypernatremic burden—and investigated their associations with 30-day mortality, DCI, delayed neurological deficit (DND), neurological outcome, ICU length of stay, and early brain edema assessed by SEBES. We hypothesized that longitudinal sodium indices would provide greater prognostic value than admission sodium concentration alone and would be associated with markers of EBI. To place our findings in the context of current evidence, we additionally performed a focused narrative review of studies published during the past decade.

## 2. Materials and Methods

### 2.1. Study Design and Patient Population

This retrospective observational study screened 323 consecutive adult patients with angiographically confirmed aSAH admitted to the Neurocritical Care Unit of the University Hospital in Wroclaw, Poland, between January 2014 and December 2025. After application of the predefined exclusion criteria, 91 patients were excluded, resulting in a final study cohort of 232 patients. Patients admitted more than 24 h after ictus (*n* = 23) were excluded to ensure a comparable early post-hemorrhage baseline and to minimize heterogeneity related to pre-admission fluid therapy and evolving neurological complications that could affect subsequent serum sodium trajectories. Patients with evidence of irreversible brain injury at presentation (*n* = 24), pre-existing central nervous system disease (*n* = 15), medical conditions or chronic medications likely to substantially affect serum sodium homeostasis (*n* = 15; e.g., chronic kidney disease requiring dialysis, adrenal insufficiency, chronic SIADH, or long-term diuretic therapy), immunosuppressive therapy (*n* = 4), intraoperative complications (*n* = 4), pregnancy (*n* = 3), or incomplete clinical, radiological, or laboratory data precluding longitudinal sodium analyses (*n* = 3) were also excluded.

The study was conducted in accordance with the Declaration of Helsinki. Following review of the study documentation and protocol, the Bioethics Committee of Wroclaw Medical University determined that, owing to the retrospective design of the study and the use of anonymized data, formal ethical approval and informed consent were not required (KB-162/2026; 20 July 2026). This study is reported in accordance with the STROBE statement (Strengthening the Reporting of Observational Studies in Epidemiology); https://www.strobe-statement.org/ (accessed on 13 September 2026).

### 2.2. Clinical Variables and Outcomes

Demographic characteristics, vascular risk factors, aneurysm location, treatment modality, laboratory data and clinical outcomes were extracted from the electronic medical record system.

Neurological severity at admission was assessed using the Glasgow Coma Scale (GCS), Full Outline of UnResponsiveness (FOUR) score, Hunt–Hess grade, World Federation of Neurosurgical Societies (WFNS) grade, modified Fisher grade, and SEBES.

The primary outcome was 30-day all-cause mortality. Secondary outcomes included DCI, DND, neurological status at ICU discharge (GCS and FOUR score), functional outcome at hospital discharge (modified Rankin Scale–mRS and GCS), ICU and hospital length of stay (LOS), and discharge destination. DCI and DND were analyzed as two distinct endpoints. DCI was defined as a new focal neurological deficit or a decrease in GCS of ≥2 points lasting ≥ 1 h and/or a new cerebral infarction not present on imaging 24–48 h after aneurysm securing and not explained by another cause [24,25]. DND was defined as delayed neurological worsening during hospitalization due to ischemic or non-ischemic causes.

### 2.3. Assessment of Sodium Homeostasis

Admission serum sodium concentration was defined as the first available sodium measurement obtained at initial clinical presentation. All subsequent serum sodium measurements obtained during hospitalization were retrieved from the electronic laboratory database. A total of 9338 serum sodium measurements were analyzed, comprising 232 admission measurements and 9106 subsequent measurements obtained during hospitalization (median 30 [IQR 20–57] measurements per patient). When multiple sodium measurements were available on the same day, their arithmetic mean was used to derive the daily mean sodium concentration.

Hyponatremia was defined as a serum sodium concentration < 135 mmol/L, hypernatremia as >145 mmol/L, severe hyponatremia as <130 mmol/L, and severe hypernatremia as >150 mmol/L.

The following sodium-derived indices were calculated: admission serum sodium concentration, mean sodium concentration during hospitalization, overall sodium variability, interday sodium variability, mean daily sodium change, maximum daily sodium change, the number of hypo- and hypernatremic measurements, number of days with hypo- and hypernatremia, and severe hypernatremia. Overall sodium variability was defined as the standard deviation of all serum sodium measurements, for an individual patient, whereas interday sodium variability was defined as the standard deviation of daily mean sodium concentrations. These sodium-derived indices were evaluated in relation to mortality, neurological complications, functional outcome, LOS, and early brain edema.

### 2.4. Statistical Analysis

Continuous variables are presented as mean (SD; standard deviation) or median (IQR; interquartile range), as appropriate, and categorical variables as frequencies and percentages. Between-group comparisons were performed using Student’s *t* test, Mann–Whitney U test, χ^2^ test, or Fisher’s exact test, as appropriate. Correlations were assessed using Spearman’s rank correlation coefficient. Multivariable logistic, linear, and ordinal logistic regression models were used to evaluate independent associations after adjustment for clinically relevant covariates.

Longitudinal serum sodium trajectories were analyzed using linear mixed-effects models with patient-specific random intercepts. Additional mixed-effects models aligned repeated sodium measurements to the day of DCI or DND onset. Time-to-event analyses were performed using Kaplan–Meier methodology and the log-rank test.

Receiver operating characteristic (ROC) analyses were used to assess discriminative performance of sodium-derived indices and multivariable models. The area under the curve (AUC) was used as a measure of discrimination, and AUCs were compared using DeLong’s test, with false discovery rate adjustment for multiple pairwise comparisons. Incremental predictive value was assessed using likelihood-ratio tests, continuous net reclassification improvement (NRI), and integrated discrimination improvement (IDI). Model performance was further evaluated using calibration statistics, events-per-variable (EPV) assessment, and bootstrap internal validation with optimism-corrected AUC estimates. Multicollinearity was assessed using variance inflation factors.

The association between interday sodium variability and mortality was additionally examined using restricted cubic splines. To address potential immortal-time bias, a landmark analysis was performed using sodium variability calculated from serum sodium measurements obtained during the first 5 days after admission. Patients who were alive at day 5 and had sufficient sodium measurements to calculate interday variability were included in the landmark cohort, and survival was assessed from day 5 onward. A complete-case sensitivity analysis was also performed, and the proportional hazards assumption was assessed using Schoenfeld residuals.

For Kaplan–Meier analyses, patients were dichotomized according to the cohort median interday sodium variability. This cohort-derived cutoff was used for descriptive stratification and was not considered a clinically validated threshold.

All analyses were performed using R version 4.5.2 (R Foundation for Statistical Computing, Vienna, Austria) and RStudio version 2026.1.2.418 (Posit Software, Boston, MA, USA). All tests were two-sided, and *p* < 0.05 was considered statistically significant.

### 2.5. Focused Literature Review

To contextualize the findings of the cohort study, we performed a focused narrative review of contemporary evidence on dysnatremia after aSAH. PubMed, Scopus, and Web of Science were searched for English-language original articles, review articles, clinical guidelines, and consensus statements published between 1 January 2016 and 24 July 2026. Search terms included combinations of aneurysmal subarachnoid hemorrhage, dysnatremia, hyponatremia, hypernatremia, cerebral salt wasting, syndrome of inappropriate antidiuretic hormone secretion, and sodium disturbances. Reference lists of eligible publications were manually screened to identify additional relevant studies. As this was a focused narrative review intended to provide contemporary clinical context, no formal systematic review methodology or quantitative synthesis was performed.

## 3. Results

### 3.1. Patient Characteristics

A total of 232 consecutive patients with aSAH were included in the study. Baseline demographic, clinical, laboratory, and treatment characteristics are presented in Table 1. The median age was 56 years (IQR 46–67), 57.8% of patients were women, and endovascular coiling was the predominant treatment modality (69.8%). Nearly half of the patients presented with poor-grade hemorrhage, with 47.0% classified as Hunt–Hess IV–V and 47.0% as WFNS IV–V, while 83.6% had an extensive blood burden (modified Fisher grade III–IV).

### 3.2. Sodium Dynamics During Hospitalization

At hospital admission, most patients were normonatremic (83.2%), whereas 11.2% presented with hyponatremia and 5.6% with hypernatremia. During hospitalization, patients underwent a median of 30 serum sodium measurements (mean, 40) over a median hospital stay of 8 days. Hyponatremia and hypernatremia occurred at any time during hospitalization in 58.2% and 56.0% of patients, respectively. Severe hyponatremia and severe hypernatremia occurred in 20.3% and 32.3% of patients, respectively. The median mean serum sodium concentration during hospitalization was 140.0 mmol/L (IQR 137.5–143.2). Median overall sodium variability and interday sodium variability were 3.57 mmol/L (IQR 2.49–5.13) and 3.23 mmol/L (IQR 2.13–4.84), respectively.

### 3.3. Association Between Sodium Dynamics and 30-Day Mortality

Patients who died within 30 days exhibited significantly greater disturbances in sodium homeostasis than survivors. Compared with survivors, non-survivors had higher mean serum sodium concentrations, greater overall and interday sodium variability, larger day-to-day sodium fluctuations, and a markedly greater hypernatremia burden, whereas the between-group difference in admission sodium concentration was less pronounced (Table 2). Severe hypernatremia (>150 mmol/L) occurred in 81.2% of non-survivors compared with 19.6% of survivors (*p* < 0.001, Table 2). Although admission sodium concentration differed significantly between groups, its discriminatory ability for 30-day mortality was limited (AUC 0.606, 95% CI 0.509–0.703).

Among sodium-derived variables, intraday maximum sodium demonstrated the highest discriminatory performance for 30-day mortality (AUC 0.901, 95% CI 0.851–0.951). Other measures with high discriminatory performance included interday mean sodium (AUC 0.894, 95% CI 0.843–0.944), interday maximum sodium (AUC 0.889, 95% CI 0.832–0.945), mean serum sodium concentration (AUC 0.887, 95% CI 0.836–0.939), and maximum sodium concentration (AUC 0.879, 95% CI 0.820–0.938). Measures of sodium variability also demonstrated discriminatory ability: interday sodium variability had an AUC of 0.816 (95% CI 0.735–0.896), whereas mean daily change and maximum daily change yielded AUCs of 0.808 and 0.802, respectively. Baseline characteristics according to low and high interday sodium variability are presented in Supplementary Table S1. In contrast, measures based on sodium range or within-day variability generally showed lower discriminatory performance. Detailed ROC analyses of individual sodium-derived indices are provided in Supplementary Table S2. Pairwise comparisons between interday sodium variability and the other sodium-derived indices are presented in Supplementary Table S3. The sodium-derived indices showed substantial correlations, particularly among measures describing related dimensions of sodium variability (Supplementary Figure S1).

In multivariable logistic regression, higher interday sodium variability remained independently associated with 30-day mortality after adjustment for age, sex, WFNS grade, modified Fisher grade, SEBES, and admission glucose (OR 1.55, 95% CI 1.29–1.86; *p* < 0.001). In the fully adjusted model, which additionally included severe hypernatremia, interday sodium variability remained independently associated with mortality (OR 1.32, 95% CI 1.09–1.60; *p* = 0.005), while severe hypernatremia was also independently associated with mortality (OR 4.66, 95% CI 1.57–13.77; *p* = 0.005).

**Figure 1:**
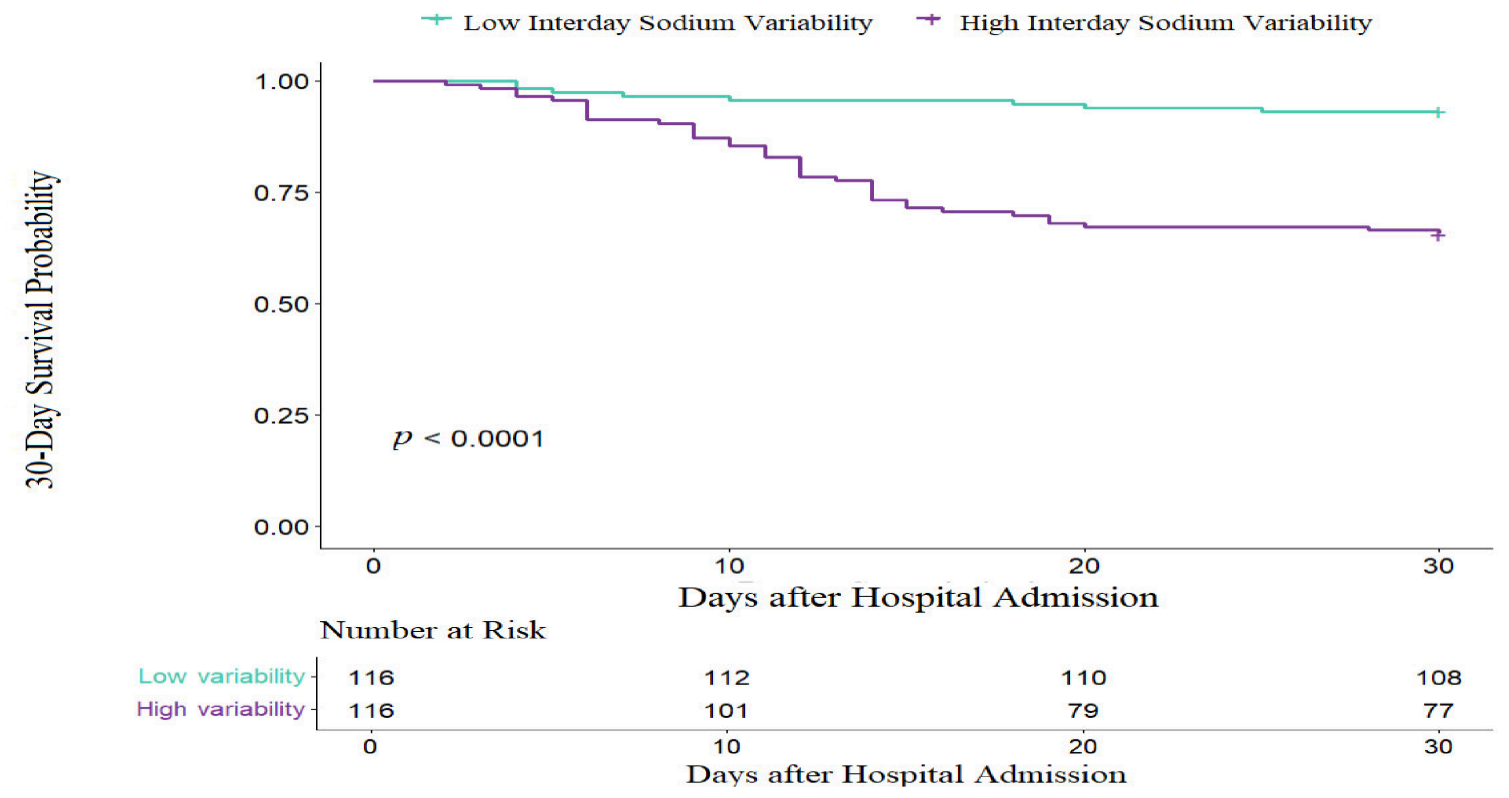
Kaplan–Meier curves for 30-day survival according to interday sodium variability. Patients were stratified using the cohort median interday sodium standard deviation (SD) of 3.23 mmol/L into lower (≤3.23 mmol/L) and higher (>3.23 mmol/L) variability groups. The cutoff was derived from the study cohort and is not intended to represent a clinically validated threshold. Patients with higher interday sodium variability had significantly lower 30-day survival (log-rank *p* < 0.001).

Patients with higher interday sodium variability had significantly lower 30-day survival (log-rank *p* < 0.001; Figure 1). The baseline, interday variability, and fully adjusted models demonstrated AUCs of 0.845 (95% CI 0.790–0.900), 0.899 (95% CI 0.851–0.947), and 0.918 (95% CI 0.880–0.956), respectively. Compared with the baseline model, addition of interday sodium variability significantly improved discrimination (DeLong test, ΔAUC = 0.054, 95% CI 0.010–0.099; *p* = 0.017). Addition of severe hypernatremia to the interday variability model did not significantly increase the AUC (ΔAUC = 0.019, 95% CI −0.006–0.043; *p* = 0.133), whereas the fully adjusted model demonstrated significantly better discrimination than the baseline model (ΔAUC = 0.073, 95% CI 0.025–0.121; *p* = 0.003). Likelihood-ratio tests similarly supported the incremental contribution of interday sodium variability (χ^2^ = 34.49, *p* < 0.001) and severe hypernatremia (χ^2^ = 7.82, *p* = 0.005). Addition of severe hypernatremia to the interday variability model yielded a continuous NRI of 0.737 (95% CI 0.316–1.202) and an IDI of 0.178 (95% CI 0.064–0.319).

**Figure 2:**
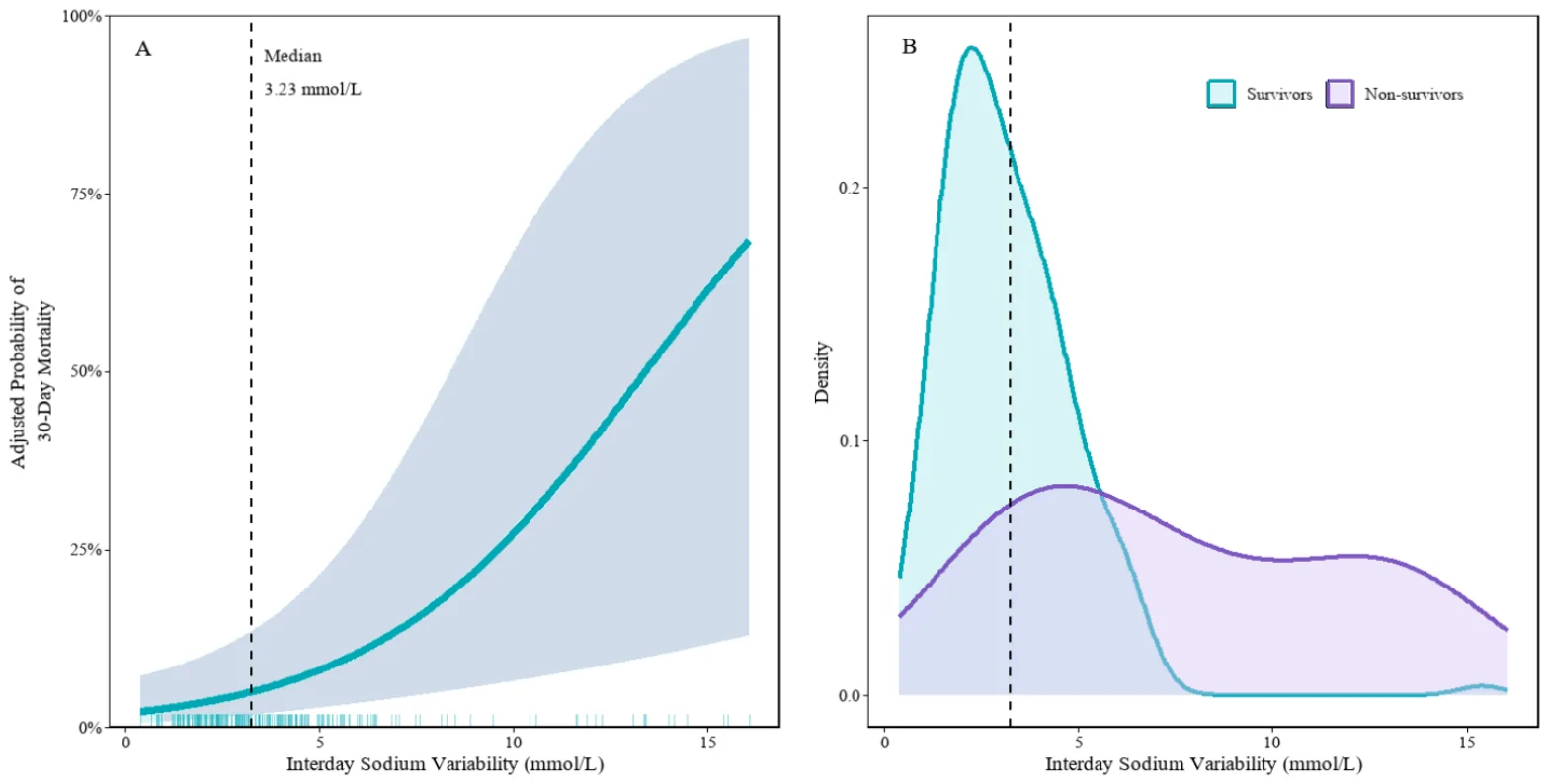
Association between interday sodium variability and 30-day mortality. (**A**) Adjusted predicted probability of 30-day mortality according to interday sodium variability derived from the final multivariable logistic regression model adjusted for age, sex, WFNS grade, modified Fisher grade, Subarachnoid Hemorrhage Early Brain Edema Score (SEBES), admission glucose concentration, and severe hypernatremia. The shaded area represents the 95% confidence interval (CI). The vertical dashed line indicates the cohort median interday sodium variability (3.23 mmol/L). (**B**) Distribution of interday sodium variability among survivors and non-survivors. Density curves represent the distribution of interday sodium variability, and rug plots indicate individual patient observations.

The adjusted predicted probability of 30-day mortality across the range of interday sodium variability, together with the distribution of interday sodium variability according to mortality status, is shown in Figure 2. Model performance was further supported by favorable Brier scores (0.123, 0.093, and 0.090 for the three models, respectively) and non-significant Hosmer–Lemeshow tests (*p* = 0.961, 0.957, and 0.565, respectively). The estimated events per variable were 8.0 for the baseline model, 6.9 for the interday variability model, and 6.0 for the fully adjusted model. Bootstrap internal validation yielded optimism-corrected AUCs of 0.823, 0.880, and 0.897, respectively.

The relationship between interday sodium variability and mortality was further assessed using restricted cubic splines (Supplementary Figure S2). Interday sodium variability showed a significant overall association with mortality (Wald χ^2^ = 9.57, df = 3, *p* = 0.023), whereas the nonlinear component was not statistically significant (χ^2^ = 4.16, df = 2, *p* = 0.125), providing no strong evidence for a nonlinear association.

To address potential immortal-time bias, a landmark analysis restricted the assessment of sodium variability to measurements obtained during the first 5 days after admission, with mortality evaluated thereafter. In the adjusted landmark analysis, interday sodium variability remained independently associated with subsequent mortality (HR 1.21, 95% CI 1.13–1.29; *p* < 0.001). A complete-case sensitivity analysis using the available first 5-day sodium data yielded a similar estimate (HR 1.19, 95% CI 1.11–1.28; *p* < 0.001). The global proportional hazards assumption was not violated (*p* = 0.592).

### 3.4. Temporal Sodium Trajectories and Neurological Complications

Patients who developed DCI had higher mean serum sodium concentrations (141.0 vs. 139.4 mmol/L), greater overall sodium variability (4.0 vs. 3.1 mmol/L), and greater interday sodium variability (4.0 vs. 2.9 mmol/L) than those without DCI (all *p* ≤ 0.003). In contrast, admission sodium concentration (*p* = 0.500) and mean daily sodium change (*p* = 0.093) did not differ significantly between groups. Similar findings were observed among patients who developed DND, who had higher mean serum sodium concentrations (141.6 vs. 139.1 mmol/L), greater overall sodium variability (4.5 vs. 3.0 mmol/L), greater interday sodium variability (4.2 vs. 2.8 mmol/L), and a greater hypernatremia burden (all *p* ≤ 0.001) (Table 2).

In multivariable analyses, interday sodium variability was not independently associated with DCI (OR 1.03, 95% CI 0.93–1.13; *p* = 0.624). In contrast, greater interday sodium variability was independently associated with DND (OR 1.13, 95% CI 1.02–1.27; *p* = 0.029) after adjustment for age, sex, admission WFNS grade, modified Fisher grade, and admission glucose concentration (Table 3).

To further characterize differences in serum sodium trajectories according to neurological complications, longitudinal serum sodium trajectories were analyzed over time. The time × DCI interaction was significant (β = 0.199 mmol/L/day, 95% CI 0.120–0.279; *p* < 0.001), indicating that the temporal pattern of serum sodium concentrations differed between patients with and without DCI. Similarly, the time × DND interaction was significant (β = 0.136 mmol/L/day, 95% CI 0.056–0.216; *p* < 0.001), indicating different temporal sodium trajectories between patients with and without DND.

**Figure 3:**
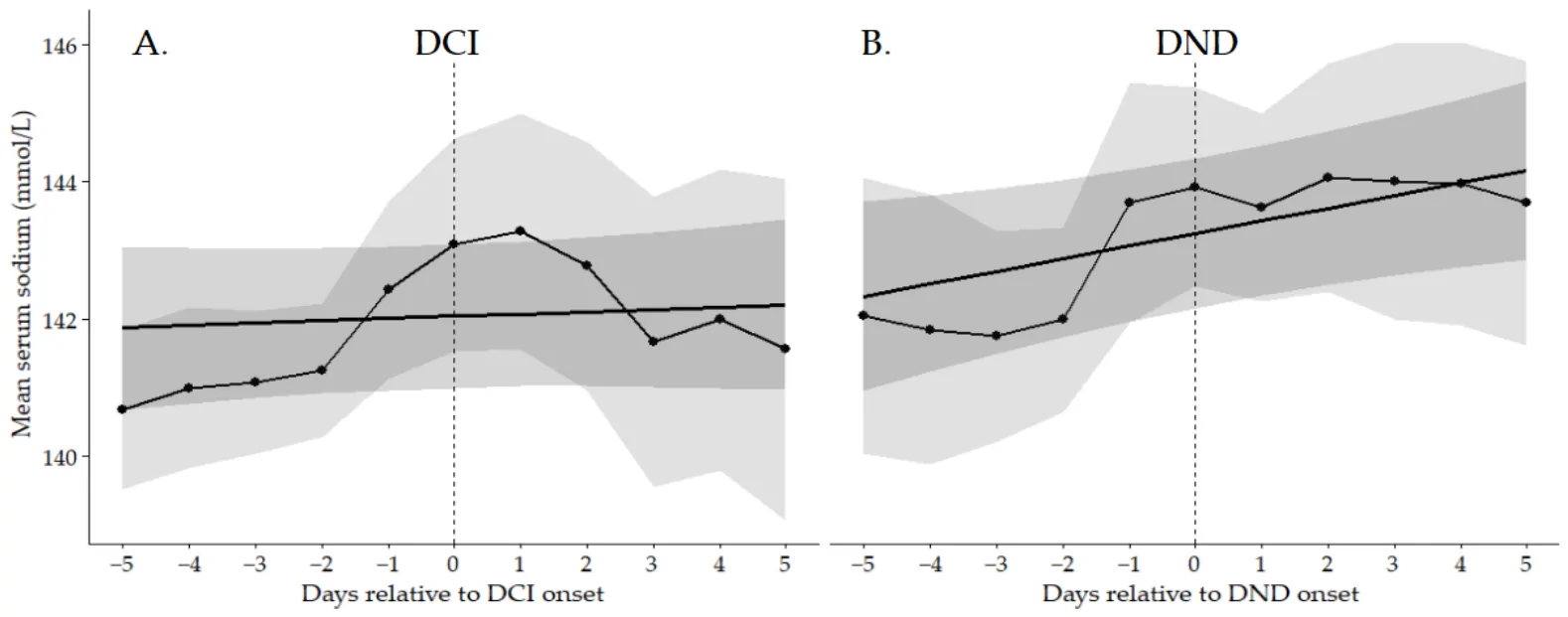
Temporal evolution of mean serum sodium concentration from five days before to five days after secondary neurological complication onset. (**A**) Sodium trajectory preceding delayed cerebral ischemia (DCI). (**B**) Sodium trajectory preceding delayed neurological deficit (DND). Values were estimated using linear mixed-effects models. The vertical dashed line indicates the day of complication onset (day 0). Shaded areas represent 95% confidence intervals (CI).

In a separate analysis restricted to patients who developed DCI or DND, repeated serum sodium measurements were aligned to the day of complication onset and analyzed from five days before to five days after the event (Figure 3). During the five days preceding DCI, serum sodium concentrations increased significantly among patients who subsequently developed DCI (β = 0.388 mmol/L/day, 95% CI 0.126–0.649; *p* = 0.004). Similarly, patients who developed DND exhibited a significant increase in serum sodium concentrations during the five days preceding neurological deterioration (β = 0.637 mmol/L/day, 95% CI 0.248–1.025; *p* = 0.002). The corresponding temporal trajectories and 95% confidence intervals are shown in Figure 3.

### 3.5. Sodium Variability and Neurological Outcome

**Figure 4:**
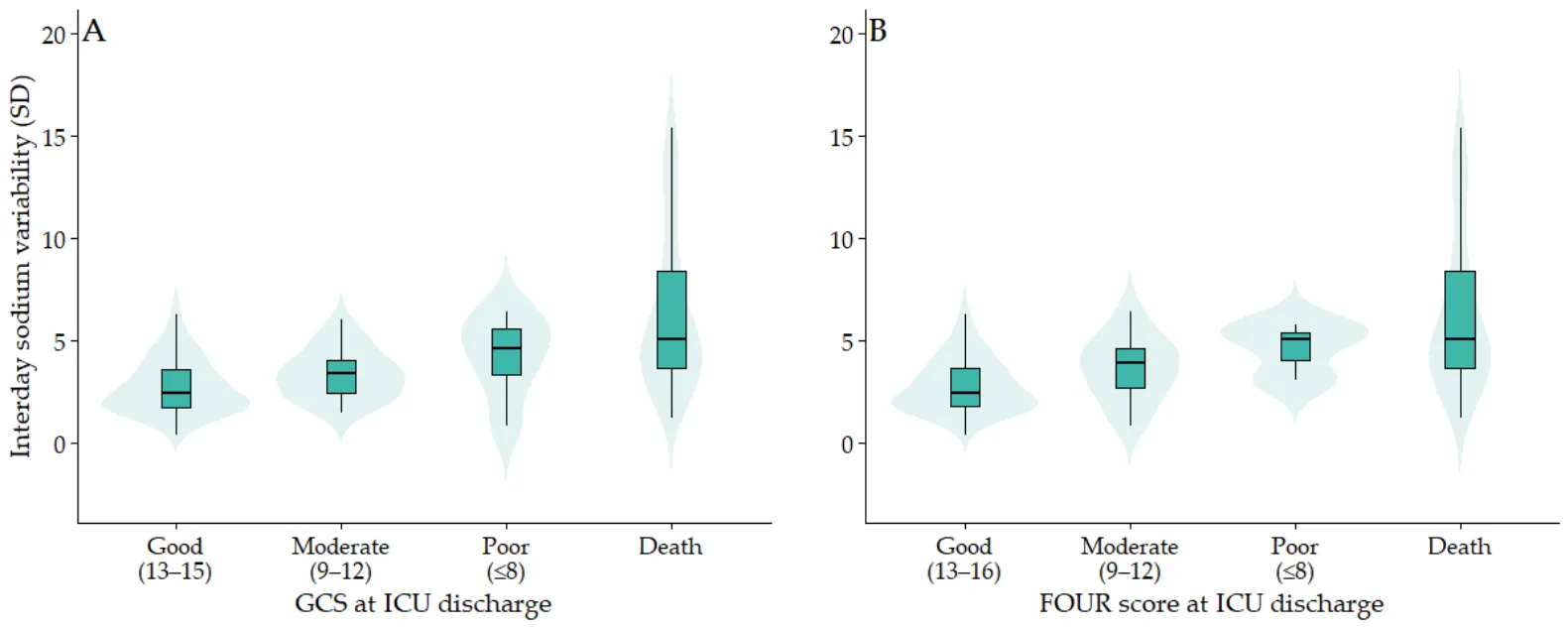
Association between interday sodium variability and neurological status at intensive care unit (ICU) discharge. (**A**) Interday sodium variability according to Glasgow Coma Scale (GCS) categories: good (13–15), moderate (9–12), poor (≤8), and death. (**B**) Interday sodium variability according to Full Outline of UnResponsiveness (FOUR) score categories: good (13–16), moderate (9–12), poor (≤8), and death. Violin plots illustrate data distributions; boxplots indicate the median, interquartile range (IQR), and 1.5 × IQR whiskers.

**Figure 5:**
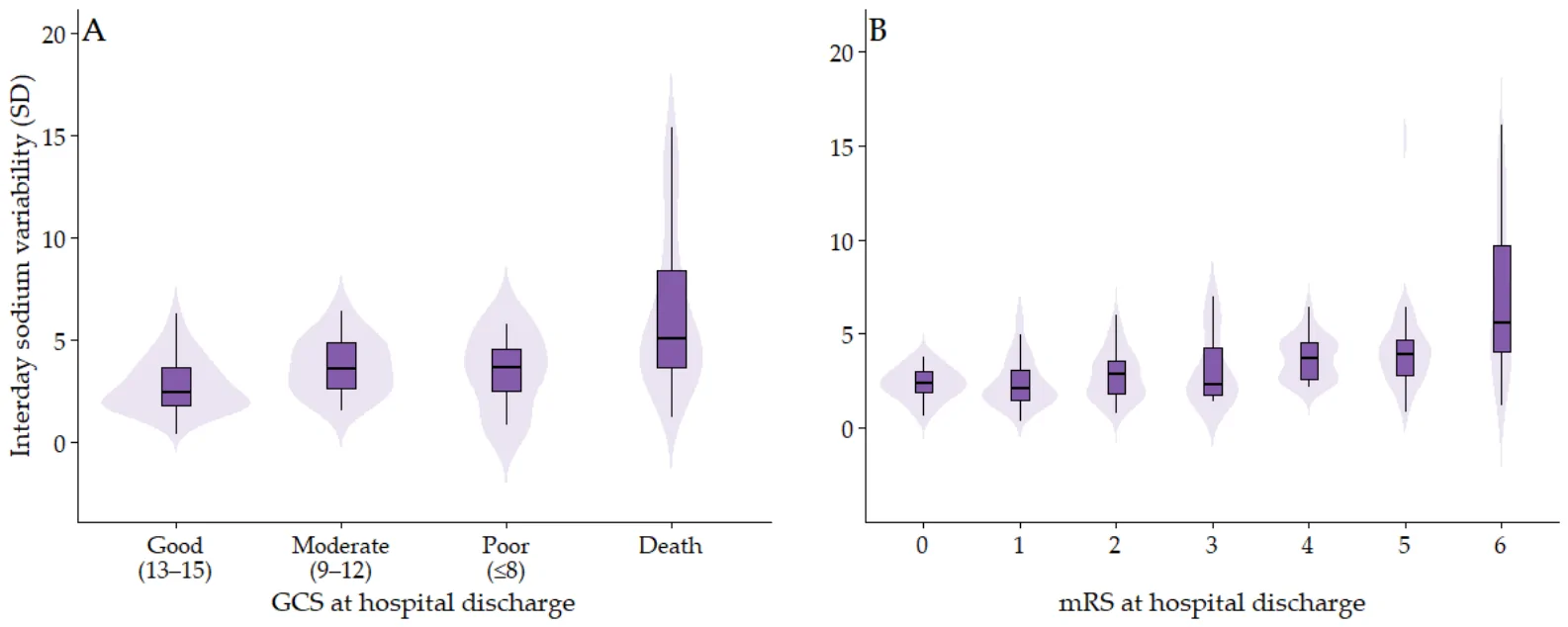
Association between interday sodium variability and neurological outcome at hospital discharge. (**A**) Interday sodium variability according to Glasgow Coma Scale (GCS) categories: good (13–15), moderate (9–12), poor (≤8), and death. (**B**) Interday sodium variability according to modified Rankin Scale (mRS) scores (0–6). Violin plots illustrate data distributions; boxplots indicate the median, interquartile range (IQR), and 1.5 × IQR whiskers.

Greater interday sodium variability was independently associated with poorer neurological outcomes at ICU and hospital discharge. Specifically, higher interday sodium variability was associated with lower GCS and FOUR scores at ICU discharge, as well as lower GCS scores and higher modified mRS scores at hospital discharge (Table 3; Figure 4 and Figure 5).

In analyses of the entire cohort, greater interday sodium variability was associated with shorter ICU and hospital LOS (Table 3). When the analysis was restricted to hospital survivors, the association was reversed: greater interday sodium variability was independently associated with longer ICU stay (β = 1.07, 95% CI 0.19–1.95; *p* = 0.017) and hospital stay (β = 3.69, 95% CI 1.33–6.05; *p* = 0.002).

Patients with greater interday sodium variability were less frequently discharged home and more frequently required transfer to rehabilitation or long-term care facilities.

### 3.6. Sodium Variability and Early Brain Injury

Higher SEBESs were associated with greater neurological severity at admission, including higher WFNS (ρ = 0.357, *p* < 0.001) and Hunt–Hess grades (ρ = 0.355, *p* < 0.001), as well as lower admission GCS (ρ = −0.341, *p* < 0.001) and FOUR scores (ρ = −0.351, *p* < 0.001). Higher SEBESs were also associated with higher mean serum sodium concentration (ρ = 0.315, *p* < 0.001), greater interday sodium variability (ρ = 0.261, *p* < 0.001), greater interday sodium range (ρ = 0.274, *p* < 0.001), and a higher number of days with hypernatremia (ρ = 0.241, *p* < 0.001). Higher SEBES was additionally associated with poorer neurological status at ICU and hospital discharge, including lower GCS at ICU discharge (ρ = −0.319, *p* < 0.001) and higher mRS at hospital discharge (ρ = 0.275, *p* < 0.001).

However, SEBES was not independently associated with 30-day mortality after adjustment for age, sex, admission WFNS grade, modified Fisher grade, and admission glucose concentration (OR 1.08, 95% CI 0.83–1.40; *p* = 0.572). This association remained non-significant after additional adjustment for interday sodium variability (OR 0.96, 95% CI 0.71–1.30; *p* = 0.799) or severe hypernatremia (OR 1.04, 95% CI 0.76–1.42; *p* = 0.810). In contrast, interday sodium variability remained independently associated with 30-day mortality when included in the model (OR 1.55, 95% CI 1.29–1.86; *p* < 0.001), as did severe hypernatremia (OR 11.33, 95% CI 4.51–28.55; *p* < 0.001).

### 3.7. Additional Analyses

In exploratory analyses, active smoking was independently associated with lower admission serum sodium concentration (β = −1.59 mmol/L, 95% CI −2.99–−0.18; *p* = 0.027), whereas posterior circulation aneurysm location was independently associated with higher admission serum sodium concentration (β = 3.01 mmol/L, 95% CI 1.26–4.76; *p* < 0.001). In a multivariable logistic regression model, higher admission serum sodium concentration was independently associated with increased 30-day mortality after adjustment for age, sex, admission WFNS grade, modified Fisher grade, glucose concentration, and ischemic heart disease (OR 1.13, 95% CI 1.04–1.24; *p* = 0.005).

Sex-related differences were limited. Women more frequently had multiple intracranial aneurysms (33.6% vs. 16.3%; *p* = 0.004) and arterial hypertension (53.0% vs. 38.8%; *p* = 0.034), whereas current smoking was more common among men (72.4% vs. 40.3%; *p* < 0.001). No significant sex differences were observed in DCI, DND, 30-day mortality, treatment modality, posterior circulation aneurysm location, ischemic heart disease, or the occurrence of hypo- or hypernatremia. Sex was not independently associated with interday sodium variability (β = −0.31, 95% CI −1.07–0.45; *p* = 0.425). Furthermore, there was no significant interaction between sex and interday sodium variability in relation to 30-day mortality (β = −0.19, 95% CI −0.64–0.20; *p* = 0.370).

Sensitivity analyses supported the robustness of the association between interday sodium variability and 30-day mortality. The association remained statistically significant among patients with observation periods of at least 7 days (OR 1.73, 95% CI 1.32–2.26; *p* < 0.001), 10 days (OR 1.56, 95% CI 1.19–2.04; *p* = 0.001), and 14 days (OR 1.87, 95% CI 1.20–2.91; *p* = 0.006). The association also remained significant after additional adjustment for the number of serum sodium measurements and duration of observation (OR 1.79, 95% CI 1.38–2.32; *p* < 0.001).

To further address potential immortal-time bias, a landmark analysis restricted sodium variability assessment to the first 5 days after admission was performed, with mortality evaluated thereafter. In the univariable landmark analysis, interday sodium variability was associated with subsequent mortality (HR 1.24, 95% CI 1.17–1.32; *p* < 0.001). After adjustment for age and WFNS grade, the association remained significant (HR 1.19, 95% CI 1.11–1.28; *p* < 0.001). The global proportional hazards assumption was not violated (*p* = 0.592).

## 4. Discussion

To place our findings in the context of the current literature, we performed a focused narrative review of studies published between 2016 and 2026. The review primarily included publications from the preceding decade to reflect current neurocritical care practice, diagnostic approaches, and therapeutic strategies. Earlier landmark studies were additionally incorporated where appropriate to provide historical perspective and to outline the fundamental concepts related to hyponatremia, hypernatremia, SIADH, and CSW. The characteristics and principal findings of the included studies are summarized in Table 4.

### 4.1. Principal Findings

The present study demonstrates that longitudinal sodium dynamics provide additional prognostic information beyond isolated admission serum sodium concentration following aSAH. Consistent with recent reports, admission sodium concentration showed limited prognostic utility, whereas greater interday sodium variability was independently associated with 30-day mortality and unfavorable neurological outcomes after adjustment for established markers of disease severity [14,19,20,21]. Adding interday sodium variability to the baseline clinical model significantly improved discrimination for 30-day mortality, increasing the AUC from 0.845 to 0.899. This association remained significant in sensitivity analyses and in a landmark analysis restricted to sodium variability assessed during the first 5 days after admission, supporting the robustness of the finding despite the potential for time-related bias.

Greater interday sodium variability was also associated with poorer neurological status at ICU and hospital discharge, as well as with early brain edema and longer ICU and hospital stay among hospital survivors. Although patients who developed DCI or DND exhibited distinct sodium trajectories, the independent association with DCI was not retained after multivariable adjustment, whereas interday sodium variability remained independently associated with DND. Taken together, these findings suggest that longitudinal disturbances in sodium homeostasis may serve as clinically relevant markers of evolving disease severity and neurological deterioration after aSAH, rather than representing isolated electrolyte abnormalities or necessarily causal mechanisms of adverse outcomes.

### 4.2. Dysnatremia After aSAH: Incidence and Prognostic Significance

Dysnatremia remains one of the most frequent metabolic complications following aSAH, affecting approximately 30–55% of patients during the acute phase, although reported incidence varies according to patient characteristics, diagnostic definitions, timing of sodium assessment, and monitoring protocols [1,4]. These disturbances occur within the complex pathophysiological cascade of early brain injury (EBI) and subsequent systemic responses that characterize the acute phase of aSAH [1,2]. A large PRISMA-compliant meta-analysis including 52 studies and more than 10,000 patients demonstrated that hyponatremia is associated with an increased risk of cerebral vasospasm and prolonged hospitalization, but not with poor functional outcome after adjustment for confounding factors [4]. Variability among published studies may reflect differences in patient age, baseline neurological severity, and, importantly, the distinction between admission and delayed-onset hyponatremia, which may represent biologically distinct processes with different clinical implications [9,26].

Hyponatremia has traditionally received the greatest clinical attention because it commonly results from cerebral salt wasting (CSW) or syndrome of inappropriate antidiuretic hormone secretion (SIADH) and has been associated with prolonged hospitalization and neurological complications [5,7]. Earlier observational studies and the systematic review by Mapa et al. suggested associations between hyponatremia, cerebral vasospasm, and prolonged hospitalization, but not mortality [8,29]. However, accumulating evidence indicates that hyponatremia itself is rarely an independent determinant of mortality or poor functional outcome once disease severity and other clinical confounders are considered [14,15,20,30].

Our findings are consistent with this broader perspective. Dysnatremia was common throughout hospitalization, with both hyponatremia and hypernatremia occurring in more than half of the cohort. Although admission serum sodium concentration was independently associated with 30-day mortality after adjustment for clinical factors, its discriminatory performance as an individual predictor was limited (AUC 0.606). In contrast, longitudinal sodium measures, particularly interday sodium variability, provided additional prognostic information beyond admission sodium concentration. Contemporary evidence also suggests that hypernatremia, higher mean sodium concentrations, and greater sodium variability may show more consistent associations with adverse outcomes than hyponatremia alone [14,17,21]. These findings support a broader assessment of sodium homeostasis that considers both the magnitude and temporal evolution of sodium disturbances rather than focusing exclusively on isolated episodes of hyponatremia.

### 4.3. Beyond Hyponatremia: The Importance of Sodium Trajectories

Growing evidence indicates that the prognostic significance of dysnatremia extends beyond isolated episodes of hypo- or hypernatremia and may be better characterized by longitudinal sodium trajectories [14,19,20,21]. Early observational studies by Bales et al. suggested that sodium variability was associated with neurological outcome, highlighting the potential importance of dynamic rather than static sodium assessment [19]. Similarly, Okazaki et al. demonstrated that both minimum and maximum serum sodium concentrations were independently associated with unfavorable neurological outcomes, suggesting that both lower and higher sodium concentrations may be relevant to prognosis after aSAH [12]. This concept was subsequently reinforced by Eagles et al., who reported that greater day-to-day deviation from baseline sodium concentrations was associated with unfavorable functional outcome following aSAH [28]. Cohen et al. further strengthened this concept by identifying both dysnatremia and increased sodium variability as independent predictors of poor 6-month neurological outcome in a prospective multicenter cohort. Importantly, they also demonstrated that a high admission sodium concentration followed by a gradual decline was associated with unfavorable neurological outcome, further emphasizing the potential prognostic relevance of sodium trajectories [20]. More recently, Labib et al. showed that higher mean sodium concentrations and greater sodium variability were independently associated with mortality irrespective of DCI, while Wang et al. and Uchida et al. further reported associations between sodium variability and adverse outcomes in surgical and elderly patient populations, respectively [14,21,35].

Not all studies, however, have reached similar conclusions. Chua et al. found no independent association between longitudinal sodium variability and radiographic vasospasm, neurological deterioration, functional outcome, or mortality, although higher mean sodium concentrations were observed among patients with adverse outcomes [16]. These discrepant findings likely reflect differences in study populations, sodium metrics, monitoring protocols, and extent of statistical adjustment for disease severity. Our findings are broadly consistent with the growing evidence supporting longitudinal assessment of sodium homeostasis. Interday sodium variability remained independently associated with 30-day mortality and unfavorable neurological outcomes after adjustment for established prognostic factors. Furthermore, incorporating interday sodium variability into a baseline clinical model significantly improved discrimination for mortality. Importantly, interday sodium variability was not the strongest-performing individual sodium-derived discriminator in our cohort, indicating that its prognostic relevance should not be interpreted as evidence of superiority over all other sodium metrics. Rather, our findings support the broader concept that longitudinal sodium assessment can provide prognostic information beyond isolated admission sodium concentration. These trajectories may integrate the cumulative effects of evolving secondary brain injury, neuroendocrine dysregulation, systemic physiological stress, and treatment-related factors.

### 4.4. Sodium Disturbances and Cerebral Vasospasm

The relationship between dysnatremia and DCI or CV remains controversial [4,14]. Earlier observational studies suggested that hyponatremia frequently precedes symptomatic vasospasm and may therefore represent an early clinical marker of impending neurological deterioration [27,29]. More recent investigations have similarly reported associations between lower sodium concentrations, greater sodium fluctuations, and an increased likelihood of CV requiring intervention [32,34,36]. In contrast, several contemporary cohort studies have failed to demonstrate an independent association between sodium disturbances and DCI after adjustment for established indicators of disease severity, suggesting that sodium disturbances may primarily reflect the severity of neurological injury rather than directly contribute to vasospasm development [14,20]. These findings highlight the potential importance of considering the temporal evolution of sodium concentrations rather than focusing exclusively on isolated episodes of hyponatremia.

Our findings support this latter interpretation. Although patients who developed DCI exhibited greater sodium variability and distinct longitudinal sodium trajectories, the association with DCI was no longer independent after multivariable adjustment. In contrast, interday sodium variability remained independently associated with DND. These results suggest that sodium disturbances may primarily reflect evolving neurocritical illness and, potentially, treatment-related factors rather than representing independent determinants of DCI. The heterogeneous findings across published studies may reflect differences in DCI definitions, sodium monitoring protocols, timing of laboratory assessment, and adjustment for disease severity, highlighting the need for standardized methodologies in future prospective investigations [4,5].

### 4.5. Pathophysiological Considerations

The mechanisms underlying dysnatremia after aSAH are complex and multifactorial, involving interactions among EBI, neuroendocrine dysfunction, systemic inflammation, and therapeutic interventions [1,5,6,37]. Hyponatremia is most commonly the result of SIADH or CSW, although differentiating between these entities remains challenging because of overlapping biochemical features and the absence of universally accepted diagnostic criteria [1,5,38,39]. Accurate diagnosis requires assessment of volume status, fluid balance, urine output, serum and urine osmolality, urinary sodium excretion, renal function, and ongoing therapies rather than serum sodium concentration alone [5,38,39]. Hypernatremia, in contrast, is typically related to diabetes insipidus, osmotic therapy, excessive free-water loss, or aggressive therapeutic sodium administration [5,6,37,40]. Furthermore, Harada et al. demonstrated that longitudinal sodium trajectories parallel dynamic changes in cortisol and vasopressin physiology after aSAH, supporting the concept that sodium disturbances reflect complex neuroendocrine responses rather than isolated electrolyte shifts [32].

Accordingly, serum sodium disturbances should be viewed as dynamic physiological markers integrating the effects of brain injury, systemic stress, and neurocritical care. Disease-related mechanisms—including natriuresis, hypothalamic–pituitary dysfunction, altered vasopressin secretion, and impaired renal water handling—as well as iatrogenic factors such as hypertonic saline, mannitol, fluid management, diuretics, and corticosteroids collectively shape sodium trajectories during intensive care [5,6,37,39].

This concept may also be relevant to DND. In our cohort, greater interday sodium variability remained independently associated with DND, and patients who subsequently developed DND demonstrated a significant increase in serum sodium concentrations during the five days preceding neurological deterioration. These findings suggest that dynamic sodium disturbances may accompany the evolution of secondary neurological injury. However, whether sodium variability contributes directly to neurological deterioration or instead reflects evolving disease severity, neuroendocrine disturbances, or treatment-related changes cannot be determined from our observational data. Our findings are therefore consistent with the concept that interday sodium variability reflects the evolution of critical illness rather than acting as a direct mediator of neurological injury.

### 4.6. Clinical Implications

Recognition of longitudinal changes in serum sodium as clinically relevant prognostic markers has important implications for the management of patients with aSAH. Current guidelines recommend maintaining euvolemia, identifying the underlying cause of sodium disturbances, and avoiding both hypovolemia and overly rapid correction of serum sodium rather than targeting supraphysiological sodium concentrations [1,5,38,39]. In the broader context of neurocritical care management, Labeyrie et al. [41] compared a non-interventional strategy based on induced hypertension with an interventional strategy combining distal percutaneous balloon angioplasty and intravenous milrinone as first-line treatment for DCI. The authors found no superiority of the interventional strategy in reducing radiological DCI or improving functional outcomes, underscoring the continuing uncertainty regarding optimal DCI management. In this context, serial assessment of serum sodium may provide clinically relevant prognostic information beyond a single admission measurement, particularly in patients with severe neurological injury [14,15,19,20,21] and may therefore support early risk stratification while awaiting prospective validation.

Despite the high incidence of dysnatremia following aSAH, substantial variability persists in its diagnostic evaluation and management across neurocritical care centers [7,26]. Real-world therapeutic strategies also vary considerably, with sodium supplementation, fludrocortisone, and, less commonly, vasopressin receptor antagonists being used even in patients with mild hyponatremia, reflecting the absence of standardized management protocols [31]. Current management therefore remains largely guided by institutional practice rather than high-quality comparative evidence [3,5,38]. Although fludrocortisone may reduce natriuresis and fluid requirements in selected patients with CSW, consistent improvements in neurological outcomes have not been demonstrated [3,5,33].

Our findings suggest that monitoring sodium variability in addition to overt dysnatremia may provide complementary prognostic information. Greater interday sodium variability was independently associated with mortality and unfavorable neurological outcomes and was also associated with prolonged ICU and hospital stay among survivors. However, these findings should not be interpreted as evidence that sodium variability itself is a therapeutic target. At present, management should focus on identifying the underlying mechanism of dysnatremia, maintaining stable euvolemia and normonatremia, avoiding excessive sodium fluctuations where clinically feasible, and interpreting sodium disturbances within the broader clinical context rather than as isolated therapeutic targets [1,5,38,39]. Because available evidence is largely observational and heterogeneous with respect to dysnatremia definitions, monitoring protocols, outcome measures, and adjustment strategies, standardized, well-designed multicenter prospective studies are needed to determine whether strategies aimed at reducing sodium variability can improve clinical outcomes [3,4,7].

### 4.7. Sodium Variability and Early Brain Edema (SEBES)

Early brain edema is a hallmark of EBI after aSAH and is consistently associated with neurological deterioration, DCI, and unfavorable functional outcome [22,23]. In the present study, greater interday sodium variability was associated with higher SEBES values, suggesting that longitudinal disturbances in sodium homeostasis may accompany more severe EBI rather than merely represent isolated electrolyte abnormalities.

This association is likely multifactorial and should be interpreted cautiously. Severe brain injury may disrupt hypothalamic–pituitary and other neuroendocrine pathways, activate systemic inflammatory responses, and impair water and sodium homeostasis. At the same time, patients with higher SEBESs and more pronounced cerebral edema may be more likely to receive osmotherapy, including mannitol or HTS, which may influence serum sodium concentrations and contribute to greater interday sodium variability [1,6,38]. Thus, the observed association may reflect a combination of the severity of the underlying brain injury, physiological dysregulation, and treatment intensity.

Clinical data support the use of hyperosmolar therapy for the management of intracranial hypertension, although the optimal agent, concentration, dose, and administration strategy in aSAH remain uncertain. Koenig et al. reported reversal of transtentorial herniation following administration of 23.4% saline in a retrospective cohort of neurocritically ill patients, including 16 patients with SAH [42]. In a study specifically involving patients with aSAH, Huang and Yang found that both 3% HTS and 20% mannitol rapidly reduced ICP, with no significant difference in the magnitude or duration of ICP reduction between the two treatments [43]. A contemporary review of HTS use in neurocritical care further highlighted substantial heterogeneity in formulation, dosing, administration, and monitoring, despite an overall manageable safety profile when serum sodium and chloride concentrations are appropriately monitored [44]. The ongoing multicenter OSMO-SAH study was designed to prospectively investigate hyperosmolar therapy in severe aSAH and may provide further evidence regarding the comparative effectiveness and safety of 20% mannitol and 10% HTS [45].

Accordingly, the observed association between SEBES and sodium variability may reflect a combination of the severity of the underlying brain injury, physiological dysregulation, and treatment intensity. This raises the possibility of confounding by indication and precludes causal interpretation of the relationship between SEBES and sodium variability. Interday sodium variability may therefore integrate information related both to the severity of EBI and to the intensity of subsequent neurocritical care. Few studies have directly examined the relationship between longitudinal sodium dynamics and radiographic markers of early brain edema. Prospective multicenter studies incorporating serial sodium measurements, detailed exposure to osmotherapy, ICP monitoring, SEBES assessment, and standardized neuroimaging are therefore warranted. Such studies may help determine whether sodium variability provides prognostic information independent of treatment-related effects or partly reflects the intensity of hyperosmolar therapy.

### 4.8. Strengths and Limitations

This study has several strengths. It provides a comprehensive evaluation of sodium disturbances after aSAH by integrating conventional sodium measurements with longitudinal sodium indices and assessing their associations with mortality, neurological deterioration, functional outcome, early brain edema, and DCI. Importantly, the analyses incorporated multivariable adjustment for established markers of disease severity, formal assessment of model discrimination and calibration, and internal bootstrap validation. The robustness of the association between interday sodium variability and 30-day mortality was further supported by multiple sensitivity analyses and a landmark analysis addressing potential immortal-time bias.

Several limitations should also be acknowledged. First, this was a retrospective single-center study, which may limit the generalizability of the findings and introduces the possibility of residual confounding despite multivariable adjustment. Second, serum sodium concentrations were likely influenced by therapeutic interventions, including HTS, osmotherapy, fluid management strategies, diuretics, and corticosteroids, the effects of which could not be fully quantified in the present analysis. This limitation is particularly relevant to the observed association between sodium variability and SEBES, as patients with more severe EBI may be more likely to receive intensive osmotherapy and other interventions that alter serum sodium concentrations, resulting in potential confounding by indication. Therefore, the association between SEBES and sodium variability cannot be interpreted as evidence of a direct pathophysiological relationship.

Third, the underlying mechanisms of dysnatremia, including differentiation between SIADH, CSW, and diabetes insipidus, could not be consistently distinguished because of the retrospective study design. Fourth, although the landmark analysis addressed the potential overlap between exposure assessment and subsequent mortality, the primary analysis included sodium measurements collected throughout hospitalization; therefore, residual time-related bias cannot be completely excluded.

Fifth, the cohort median of 3.23 mmol/L used to classify lower versus higher interday sodium variability was derived from the present study population and should not be interpreted as a clinically validated threshold. External validation in independent cohorts is required before any threshold-based clinical application can be considered.

Finally, the observational nature of the study precludes causal inference, and sodium variability may represent both a marker of disease severity and a consequence of therapeutic interventions. Prospective multicenter studies with standardized treatment protocols, detailed treatment exposure, comprehensive neuroendocrine assessment, and serial serum sodium monitoring are therefore required to validate these findings and determine whether dynamic sodium indices can improve prognostic accuracy and guide individualized management after aSAH.

### 4.9. Conclusions

Longitudinal sodium dynamics provide additional prognostic information beyond isolated admission sodium concentration following aSAH. Greater interday sodium variability was independently associated with 30-day mortality and unfavorable neurological outcomes and was also associated with early brain edema and distinct sodium trajectories preceding neurological deterioration. The addition of interday sodium variability improved discrimination of 30-day mortality beyond established clinical predictors, while sensitivity and landmark analyses supported the robustness of this association.

These findings support the concept that dynamic sodium fluctuations may reflect the evolving pathophysiological response to brain injury and the intensity of neurocritical care rather than isolated electrolyte disturbances. Routine assessment of sodium trajectories throughout hospitalization may therefore provide clinically meaningful prognostic information beyond single sodium measurements. However, sodium variability should currently be regarded as a prognostic marker rather than a therapeutic target, as its relationship with disease severity and treatment exposure cannot be fully disentangled in a retrospective study. Future prospective multicenter studies are needed to validate these findings and determine whether incorporating longitudinal sodium indices into prognostic models can improve risk stratification and whether strategies aimed at reducing excessive sodium fluctuations can improve neurological outcomes after aSAH.

## Figures and Tables

**Table 1:** Baseline Characteristics According to 30-Day Mortality.

	Overall	30-Day Survivors	30-Day Non-Survivors	*p*
Demographics				
Age (years)	56.0 (46.0–67.0)	55.0 (45.0–65.2)	61.0 (52.5–69.2)	0.013
Female sex	134 (57.8%)	110 (59.8%)	24 (50.0%)	0.290
BMI (kg/m^2^)	26.3 (23.4–30.0)	26.8 (23.5–30.4)	24.7 (23.4–28.6)	0.111
Clinical severity				
GCS on admission	13.0 (6.0–14.0)	13.0 (7.8–15.0)	5.0 (4.0–9.5)	<0.001
FOUR score on admission	14.0 (8.0–16.0)	16.0 (10.0–16.0)	6.0 (5.0–12.5)	<0.001
Hunt–Hess IV–V	109 (47.0%)	71 (38.6%)	38 (79.2%)	<0.001
WFNS IV–V	109 (47.0%)	70 (38.0%)	39 (81.2%)	<0.001
Modified Fisher III–IV	194 (83.6%)	148 (80.4%)	46 (95.8%)	0.008
SEBES	2 (0–3)	2 (0–3)	2 (1–4)	0.011
Admission laboratory				
Sodium (mmol/L)	139.0 (136.0–141.0)	138.0 (136.0–141.0)	140.0 (137.0–143.0)	0.023
Potassium (mmol/L)	3.7 (3.3–4.1)	3.7 (3.3–4.1)	3.6 (3.3–4.1)	0.732
Glucose (mg/dL)	151.0 (125.8–188.0)	144.5 (119.0–173.2)	195.5 (159.0–237.0)	<0.001
WBC (10^9^/L)	13.6 (10.5–17.6)	13.5 (10.2–17.0)	14.2 (11.8–19.9)	0.047
Hemoglobin (g/dL)	13.9 (12.8–15.1)	13.9 (12.9–14.9)	14.6 (12.7–15.5)	0.184
CRP (mg/L)	3.2 (1.3–9.0)	2.9 (1.3–9.1)	4.3 (1.5–8.6)	0.769
Comorbidities				
Hypertension	109 (47.0%)	84 (45.7%)	25 (52.1%)	0.527
Ischemic heart disease	20 (8.6%)	11 (6.0%)	9 (18.8%)	0.012
Current smoker	125 (53.9%)	99 (53.8%)	26 (54.2%)	1.000
Diabetes mellitus	18 (7.8%)	15 (8.2%)	3 (6.2%)	1.000
Treatment				
Endovascular coiling	162 (69.8%)	131 (71.2%)	31 (64.6%)	0.476
Microsurgical clipping	68 (29.3%)	53 (28.8%)	15 (31.3%)	0.878

Abbreviations used in Table 1: BMI, body mass index; CRP, C-reactive protein; FOUR, Full Outline of UnResponsiveness; GCS, Glasgow Coma Scale; WBC, white blood cell count; WFNS, World Federation of Neurosurgical Societies.

**Table 2:** Sodium-Derived Parameters According to 30-Day Mortality.

	Overall	30-Day Survivors	30-Day Non-Survivors	*p*
Sodium concentrations				
Admission sodium (mmol/L)	139 (136–141)	138 (136–141)	140 (137–143)	0.023
Mean sodium (mmol/L)	140 (137.5–143.2)	139.1 (136.8–141.3)	145.8 (143.4–150.3)	<0.001
Sodium variability				
Overall sodium SD	3.6 (2.5–5.1)	3.2 (2.3–4.4)	6.8 (4.4–10.1)	<0.001
Interday sodium SD	3.2 (2.1–4.8)	2.9 (1.9–4.1)	7.0 (4.3–11.7)	<0.001
Interday coefficient of variation	2.3 (1.5–3.4)	2.1 (1.4–3.0)	4.8 (2.9–7.9)	<0.001
Interday sodium range	10.0 (5.8–15.2)	8.3 (5.2–12.8)	21.2 (12.4–29.2)	<0.001
Mean daily sodium change	2.1 (1.7–2.8)	2.0 (1.5–2.5)	3.1 (2.3–6.5)	<0.001
Maximum daily sodium change	5.2 (3.8–7.0)	4.8 (3.5–6.2)	10.5 (5.8–16.4)	<0.001
Hypernatremia burden				
Number of hypernatremic measurements	1 (0–11)	0 (0–5.2)	12 (6–23.5)	<0.001
Number of severe hypernatremic measurements	0 (0–2)	0 (0–0)	6 (1–11.2)	<0.001
Days with hypernatremia	1 (0–5)	0 (0–3)	5 (3–8)	<0.001
Severe hypernatremia (>150 mmol/L)	75 (32.3%)	36 (19.6%)	39 (81.2%)	<0.001

Abbreviations used in Table 2: SD, standard deviation.

**Table 3:** Multivariable Regression Analyses of the Association Between Interday Sodium Variability and Clinical Outcomes.

	Effect	95% CI	*p*
Delayed Cerebral Ischemia (DCI)	OR 1.03	0.93–1.13	0.624
Delayed Neurological Deficit (DND)	OR 1.13	1.02–1.27	0.029
30-Day Mortality	OR 1.32	1.09–1.60	0.005
ICU Length of Stay	β = −0.52	−1.00 to −0.04	0.033
Hospital Length of Stay	β = −1.67	−3.02 to −0.31	0.016
GCS at ICU Discharge	β = −0.45	−0.63 to −0.28	<0.001
FOUR Score at ICU Discharge	β = −0.33	−0.53 to −0.13	0.001
GCS at Hospital Discharge	β = −0.31	−0.51 to −0.11	0.003
mRS at Hospital Discharge	OR 1.54	1.33–1.77	<0.001
Severe Hypernatremia	OR 2.70	2.04–3.76	<0.001

Abbreviations: β, regression coefficient; CI, confidence interval; FOUR, Full Outline of UnResponsiveness score; GCS, Glasgow Coma Scale; ICU, intensive care unit; mRS, modified Rankin Scale; OR, odds ratio. β coefficients are reported for linear regression models, whereas odds ratios (ORs) are reported for logistic and ordinal logistic regression models. All multivariable models were adjusted for age, sex, admission WFNS grade, modified Fisher grade, and admission glucose concentration. The models for 30-day mortality and severe hypernatremia were additionally adjusted for SEBES; the 30-day mortality model additionally included severe hypernatremia as a covariate.

**Table 4:** Summary of contemporary studies evaluating sodium disturbances after aSAH (2016–2026).

Authors	Year	Sample Size	Design and Population	Main Findings
Primary observational and interventional studies				
See et al. [26]	2016	114	Single-center retrospective cohort of patients with aSAH; assessment of admission and delayed hyponatremia.	Increasing age was associated with lower initial sodium and delayed hyponatremia, indicating that the risk and timing of hyponatremia may vary with baseline patient characteristics.
Bales et al. [19]	2016	198	Retrospective adult aSAH cohort evaluating hyponatremia and longitudinal sodium fluctuation in relation to neurological outcome.	Sodium fluctuation, rather than hyponatremia alone, was associated with worse neurological outcome.
Okazaki et al. [12]	2017	131	Retrospective ICU cohort evaluating the association of minimum and maximum serum sodium concentrations with neurological outcome at hospital discharge.	Both hyponatremia and hypernatremia were independently associated with unfavorable neurological outcome.
Spatenkova et al. [13]	2017	344	Ten-year observational study of patients with acute SAH managed using a targeted sodium protocol.	Dysnatremia occurred in 35.8% of patients, most commonly as hyponatremia. Hypernatremia was an independent risk factor for in-hospital mortality and poor outcome.
Uozumi et al. [27]	2017	97	Retrospective observational study of patients with SAH managed with targeted euvolemia and eunatremia.	Serum sodium and osmolality decreased before symptomatic vasospasm, although sodium generally remained within the normal range.
Eagles et al. [28]	2019	413	Post hoc exploratory analysis of CONSCIOUS-1 trial data.	Greater daily deviation from baseline sodium was associated with unfavorable 3-month mRS after adjustment for age and WFNS grade; larger deviations were also linked to increased DCI risk in exploratory analyses.
Ridwan et al. [9]	2019	101	Prospective observational study with serial measurements of sodium, natriuresis, water balance, serum osmolality, and urine osmolality.	Early- and late-onset hyponatremia should be distinguished because their mechanisms and therapeutic approaches may differ.
Escamilla-Ocañas et al. [29]	2020	164	Retrospective cohort study of patients with aSAH evaluating the association between hyponatremia and cerebral vasospasm.	Hyponatremia was associated with a higher incidence of cerebral vasospasm than normonatremia (65.1% vs. 28.5%; *p* < 0.001) and preceded vasospasm by a median of 1.5 days.
Quinn et al. [30]	2020	200	Prospective single-center cohort; hyponatremia was defined as Na < 135 mmol/L and 12-month GOSe was assessed.	Hyponatremia occurred in 56% of participants and was not independently associated with favorable 12-month functional outcome after adjustment for baseline covariates.
Kieninger et al. [31]	2021	180	Retrospective ICU cohort evaluating the frequency, severity, treatment, and outcome of acute hyponatremia.	Treatment was commonly initiated even for mild hyponatremia using sodium chloride, fludrocortisone, or tolvaptan, illustrating heterogeneous real-world treatment strategies.
Cohen et al. [20]	2021	356	Prospective multicenter cohort from 11 ICUs in Australia and New Zealand.	Higher mean sodium concentration and greater sodium variability were associated with worse 6-month neurological outcome. A high initial sodium followed by gradual decline was also associated with poor outcome.
Chua et al. [16]	2022	271	Single-center retrospective aSAH cohort with repeated sodium measurements and radiographic vasospasm assessment.	Longitudinal sodium variation was not associated with later radiographic vasospasm, neurological deterioration, functional outcome, or mortality, although higher mean sodium was observed in patients with adverse outcomes.
Harada et al. [32]	2022	133	Prospective five-center cohort with daily sodium measurements and serial ACTH, cortisol, and AVP measurements over 14 days.	Sodium trajectories were linked to hormonal dynamics and symptomatic vasospasm, supporting a complex neuroendocrine basis for sodium changes after SAH.
Helliwell et al. [15]	2023	320	Retrospective cohort of consecutive patients with confirmed aSAH.	Neither hyponatremia nor hypernatremia was independently associated with 3-month functional outcome or vasospasm. Hypernatremia was associated with approximately seven additional hospital days after adjustment.
Elledge et al. [33]	2023	110	Retrospective treatment study of patients with aSAH and CSW who received fludrocortisone.	Fludrocortisone was associated with reduced urine output and lower fluid requirements to maintain euvolemia and normonatremia; neurological outcome benefit was not established.
Loan et al. [7]	2023	407	Prospective multicenter observational study of consecutive patients with spontaneous SAH admitted to neurosurgical units in the United Kingdom and Ireland.	Hyponatremia occurred in 43% of patients and was associated with prolonged hospitalization but not with worse functional outcome. Diagnostic evaluation and treatment were inconsistent.
Labib et al. [14]	2024	964	Observational cohort from a prospective aSAH registry (2011–2021).	Sodium level, hyponatremia, and sodium fluctuation were not associated with DCI occurrence. Higher sodium, hypernatremia, and greater fluctuation were associated with poor outcome independently of DCI.
Yamada et al. [34]	2025	216	Multicenter retrospective study of patients with low-grade aSAH assessing serum sodium concentrations during the first two weeks.	Lower minimum serum sodium was associated with vasospasm requiring endovascular treatment. A threshold of 133 mmol/L predicted this complication with moderate accuracy.
Wang et al. [21]	2025	563	Cohort of patients with aSAH treated by surgical clipping, including propensity-score analyses.	Greater sodium variability was associated with mortality, reinforcing the possible prognostic relevance of dynamic sodium changes in surgically treated patients.
Systematic and narrative reviews				
Mapa et al. [8]	2016	2387	Systematic review of 13 studies.	The pooled prevalence of at least mild hyponatremia was 36%. Hyponatremia was associated with vasospasm and longer hospitalization but not mortality.
Busl and Rabinstein [5]	2023	N/A	Narrative review addressing mechanisms, prevention, and treatment.	Dysnatremia is multifactorial and includes CSW, SIADH, diabetes insipidus, and iatrogenic causes. Evidence that sodium supplementation or mineralocorticoids improves neurological outcomes remains insufficient.
Gillespie et al. [4]	2025	10,512	PRISMA-compliant systematic review and meta-analysis of 52 studies.	The pooled incidence of hyponatremia was 37.0%. Hyponatremia increased vasospasm risk (OR 2.93) and length of stay but was not associated with poor functional outcome.
Mehmandoost et al. [3]	2026	1749	Systematic review and meta-analysis focused on diagnostic and management approaches.	Provides the newest synthesis of CSW versus SIADH mechanisms, diagnostic differentiation, and treatment strategies; substantial heterogeneity in incidence and management evidence was reported.

Table 4 Characteristics and main findings of selected studies on dysnatremia after aneurysmal subarachnoid hemorrhage published between 2016 and 2026. Abbreviations: ACTH, adrenocorticotropic hormone; aSAH, aneurysmal subarachnoid hemorrhage; AVP, arginine vasopressin; CSW, cerebral salt wasting; DCI, delayed cerebral ischemia; GOSe, extended Glasgow Outcome Scale; mRS, modified Rankin Scale; SIADH, syndrome of inappropriate antidiuretic hormone secretion; WFNS, World Federation of Neurosurgical Societies. Sample-size note: N/A = not applicable to a narrative review.

## Data Availability

Anonymized data may be made available from the corresponding author upon reasonable request.

## Supplementary Materials

The following supporting information can be downloaded at https://www.mdpi.com/article/10.3390/biomedicines14092081/s1. Figure S1: Spearman correlation matrix of sodium-derived indices during hospitalization. Correlation coefficients between admission sodium concentration and indices describing overall, interday, and intraday sodium levels and variability are shown. Positive correlations are shown in purple and negative correlations in blue; color intensity indicates the strength of the correlation. Values in the cells represent Spearman’s correlation coefficients (ρ), Figure S2: Restricted cubic spline showing the adjusted association between interday sodium variability and 30-day mortality. The model was adjusted for age, sex, WFNS grade, modified Fisher grade, Subarachnoid Hemorrhage Early Brain Edema Score (SEBES), admission glucose concentration, and severe hypernatremia. The solid line represents the predicted probability of 30-day mortality, and the shaded area represents the 95% confidence interval (CI). The vertical dashed line indicates the cohort median interday sodium variability (3.23 mmol/L). Overall association: *p* = 0.023; test for nonlinearity: *p* = 0.125, Table S1. Baseline characteristics according to low vs high interday sodium variability. Continuous variables are presented as median (interquartile range), and categorical variables as number (percentage). *p* values were calculated using the Wilcoxon rank-sum test for continuous variables and Fisher’s exact test for categorical variables. Patients were stratified according to the cohort median interday sodium standard deviation of 3.23 mmol/L. This cutoff was derived from the study cohort and does not represent a clinically validated threshold. BMI, body mass index; CRP, C-reactive protein; FOUR, Full Outline of UnResponsiveness; GCS, Glasgow Coma Scale; SEBES, Subarachnoid Hemorrhage Early Brain Edema Score; WFNS, World Federation of Neurosurgical Societies; WBC, white blood cell count; Table S2: Diagnostic performance of individual sodium-derived indices for 30-day mortality. Discrimination was assessed using receiver operating characteristic (ROC) curve analysis. AUC, area under the ROC curve; CI, confidence interval; CV, coefficient of variation; SD, standard deviation. All analyses included 232 patients and 48 deaths, Table S3: Pairwise comparison of interday sodium variability with other sodium-derived indices. Pairwise differences in the area under the receiver operating characteristic curve (AUC) were assessed using DeLong’s test for paired ROC curves. All comparisons included 232 patients with 30-day mortality as the outcome. ΔAUC represents the difference between the AUC of the comparator index and that of interday sodium SD. *p* values were adjusted for multiple comparisons using the Benjamini–Hochberg false discovery rate (FDR) procedure. AUC, area under the receiver operating characteristic curve; CV, coefficient of variation; FDR, false discovery rate; ROC, receiver operating characteristic; SD, standard deviation. STROBE Statement—checklist of items that should be included in reports of observational studies.

## Abbreviations

ACTH	adrenocorticotropic hormone
aSAH	aneurysmal subarachnoid hemorrhage
AUC	area under the curve
AVP	arginine vasopressin
BMI	body mass index
CI	confidence interval
CRP	C-reactive protein
CSW	cerebral salt wasting
CV	cerebral vasospasm
DCI	delayed cerebral ischemia
DND	delayed neurological deficit
EBI	early brain injury
EPV	events per variable
FOUR	Full Outline of UnResponsiveness
GCS	Glasgow Coma Scale
GOSe	extended Glasgow Outcome Scale
HR	hazard ratio
HTS	hypertonic saline
ICP	intracranial pressure
ICU	intensive care unit
IDI	integrated discrimination improvement
IQR	interquartile range
LOS	length of stay
mRS	modified Rankin Scale
N/A	not applicable
NRI	net reclassification improvement
OR	odds ratio
PRISMA	Preferred Reporting Items for Systematic Reviews and Meta-Analyses
RCS	restricted cubic splines
ROC	receiver operating characteristic
SAH	subarachnoid hemorrhage
SD	standard deviation
SEBES	Subarachnoid Hemorrhage Early Brain Edema Score
SIADH	syndrome of inappropriate antidiuretic hormone secretion
STROBE	Strengthening the Reporting of Observational Studies in Epidemiology
VIF	variance inflation factor
WBC	white blood cell count
WFNS	World Federation of Neurosurgical Societies
