# Supplementary material for: Longitudinal Sodium Dynamics After Aneurysmal Subarachnoid Hemorrhage: A Single-Center Cohort Study with a Contemporary Literature Review
Source: Biomedicines. 2026 Sep 16;14(9):2081. doi: 10.3390/biomedicines14092081 (PMC13604548; doi:10.3390/biomedicines14092081)
Supplement: Supplementary file 1 [file biomedicines-14-02081-s001.zip › biomedicines-4522409-supplementary.pdf]

Supplementary Material: Longitudinal Sodium Dynamics After Aneurysmal Subarachnoid Hemorrhage: A Single-Center Cohort Study with a Contemporary Literature Review

Adrianna Lebiedzińska[1,*], Jarosław Kędziora[2], Jowita Woźniak[3], Katarzyna Smarzewska[4], Waldemar Goździk[2] and Małgorzata Burzyńska[2]

Clinical Department of Anesthesiology and Intensive Therapy, University Clinical Hospital in Wroclaw, 50-556 Wroclaw, Poland

2 Clinical Department of Anesthesiology and Intensive Therapy, Faculty of Medicine, Wroclaw Medical University, 50-367 Wroclaw, Poland

3 Clinical Department of Neurosurgery, University Centre for Neurology and Neurosurgery, Wroclaw Medical University, 50-367 Wroclaw, Poland

4 Clinical Department of Neurosurgery, Faculty of Medicine, Faculty of Medicine, Wroclaw Medical University, 50-367 Wroclaw, Poland

* Correspondence: adrianna.lebiedzinska@usk.wroc.pl;

Statistical Analysis

Continuous variables are presented as mean (standard deviation) or median (interquartile range), depending on data distribution, whereas categorical variables are presented as frequencies and percentages. Between-group comparisons were performed using Student's t test, the Mann–Whitney U test, the χ^2 test, or Fisher's exact test, as appropriate. Correlations were assessed using Spearman's rank correlation coefficient. Multivariable logistic and linear regression models were used to assess independent associations after adjustment for clinically relevant covariates. Longitudinal serum sodium trajectories were analyzed using linear mixed-effects models with patient-specific random intercepts to account for repeated measurements within individuals. Additional mixed-effects models aligned repeated sodium measurements to the day of DCI or DND onset to evaluate temporal changes preceding neurological complications. Time-to-event analyses were performed using Kaplan–Meier methodology and compared using the log-rank test. Receiver operating characteristic (ROC) analyses were used to assess the predictive performance of sodium-derived variables, with AUCs and 95% confidence intervals calculated using nonparametric methods. Pairwise comparisons of correlated ROC curves were performed using DeLong's test, with adjustment for multiple comparisons using the Benjamini–Hochberg false discovery rate procedure. The incremental discriminative value of extended models was assessed using continuous net reclassification improvement (NRI) and integrated discrimination improvement (IDI), with 95% confidence intervals estimated by bootstrap resampling. Model performance was further assessed using calibration, Brier scores, and bootstrap internal validation with 2000 resamples to estimate optimism-corrected discrimination. Multicollinearity was assessed using variance inflation factors (VIFs), and the number of events per variable (EPV) was reported to assess model complexity. The functional form of the association between interday sodium variability and 30-day mortality was evaluated using restricted cubic splines. For Kaplan–Meier analyses, patients were dichotomized according to the cohort median interday sodium variability. A landmark analysis restricted sodium variability assessment to the first 5 days after admission, with subsequent mortality evaluated from the landmark time point onward. Analyses were performed using R version 4.5.2 (R Foundation for Statistical Computing, Vienna, Austria) and RStudio version 2026.1.2.418 (Posit Software, Boston, MA, USA). All tests were two-sided, and $P < 0.05$ was considered statistically significant.

Table S1. Baseline characteristics according to low vs high interday sodium variability. Continuous variables are presented as median (interquartile range), and categorical variables as number (percentage). P values were calculated using the Wilcoxon rank-sum test for continuous variables and Fisher's exact test for categorical variables. Patients were stratified according to the cohort median interday sodium standard deviation of 3.23 mmol/L. This cutoff was derived from the study cohort and does not represent a clinically validated threshold. BMI, body mass index; CRP, C-reactive protein; FOUR, Full Outline of UnResponsiveness; GCS, Glasgow Coma Scale; SEBES, Subarachnoid Hemorrhage Early Brain Edema Score; WFNS, World Federation of Neurosurgical Societies; WBC, white blood cell count.

	Low interday sodium variability (≤3.23 mmol/L)	High interday sodium variability (>3.23 mmol/L)	*P*
**Demographics**			
Age (years)	53 (44–64)	61 (48–69)	0.004
Female sex	67 (57.8%)	67 (57.8%)	1.000
BMI (kg/m²)	27.7 (24.2–30.9)	25.0 (23.4–28.2)	0.009
**Clinical severity**			
GCS on admission	14 (9–15)	9 (5–13)	<0.001
FOUR score on admission	16 (11–16)	12 (6–16)	<0.001
Hunt–Hess grade	3 (2–4)	4 (3–5)	<0.001
WFNS grade	2 (1–4)	4 (2–5)	<0.001
Modified Fisher grade	3 (3–4)	4 (3–4)	<0.001
SEBES score	1 (0–2)	2 (0–4)	0.005
**Admission laboratory**			
Sodium (mmol/L)	139 (137–141)	139 (136–142)	0.935
Potassium (mmol/L)	3.8 (3.39–4.115)	3.66 (3.2875–4.005)	0.138
Glucose (mg/dL)	143 (117.5–171.8)	161 (136.5–203.5)	<0.001
WBC (10^9/L)	13.5 (10.5–16.6)	14.0 (10.6–19.0)	0.178
Hemoglobin (g/dL)	13.9 (12.8–15)	14.1 (12.8–15.2)	0.606
CRP (mg/L)	2.9 (1.4–7.3)	3.5 (1.3–10.0)	0.541
**Comorbidities**			
Hypertension	55 (47.4%)	54 (46.6%)	1.000
Ischemic heart disease	8 (6.9%)	12 (10.3%)	0.484
Current smoker	63 (54.3%)	62 (53.4%)	1.000
Diabetes mellitus	9 (7.8%)	9 (7.8%)	1.000
**Treatment**			
Endovascular coiling	85 (73.3%)	77 (66.4%)	0.317
Microsurgical clipping	31 (26.7%)	37 (31.9%)	0.471

Figure S1. Spearman correlation matrix of sodium-derived indices during hospitalization. Correlation coefficients between admission sodium concentration and indices describing overall, interday, and intraday sodium levels and variability are shown. Positive correlations are shown in purple and negative correlations in blue; color intensity indicates the strength of the correlation. Values in the cells represent Spearman's correlation coefficients (ϱ).

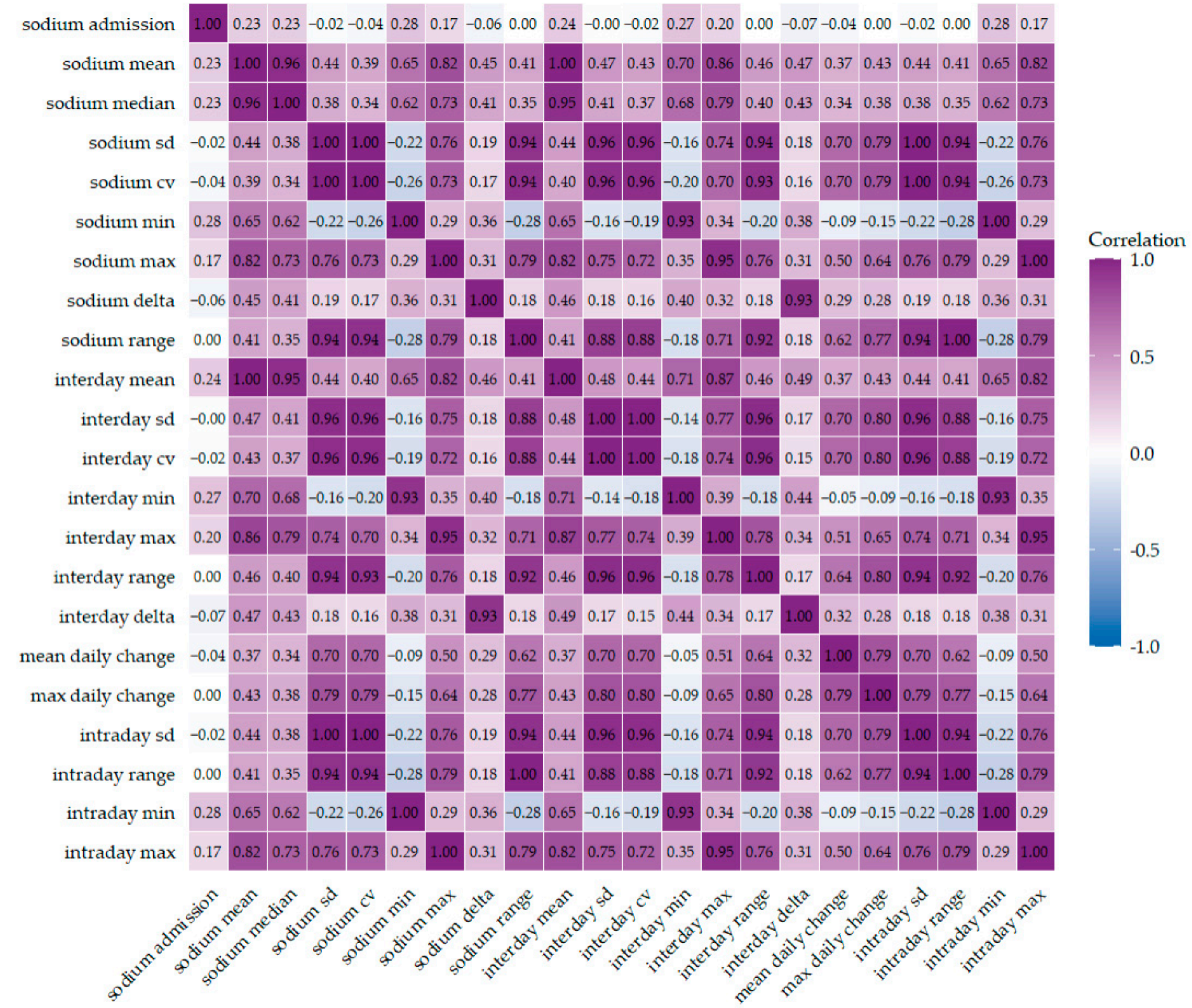

Table S2. Diagnostic performance of individual sodium-derived indices for 30-day mortality. Discrimination was assessed using receiver operating characteristic (ROC) curve analysis. AUC, area under the ROC curve; CI, confidence interval; CV, coefficient of variation; SD, standard deviation. All analyses included 232 patients and 48 deaths.

Sodium-derived index	*N*	Events	AUC	95% CI
Intraday maximum sodium	232	48	0.901	0.851–0.951
Interday mean sodium	232	48	0.894	0.843–0.944
Interday maximum sodium	232	48	0.889	0.832–0.945
Mean sodium	232	48	0.887	0.836–0.939
Maximum sodium	232	48	0.879	0.820–0.938
Intraday minimum sodium	232	48	0.872	0.811–0.933
Interday sodium delta	232	48	0.839	0.760–0.919
Sodium delta	232	48	0.831	0.748–0.914
**Interday sodium SD**	**232**	**48**	**0.816**	**0.735–0.896**
Overall sodium SD	232	48	0.813	0.734–0.891
Mean daily sodium change	232	48	0.808	0.734–0.883
Interday sodium CV	232	48	0.805	0.723–0.887
Interday sodium range	232	48	0.804	0.726–0.883
Maximum daily sodium change	232	48	0.802	0.723–0.881
Overall sodium CV	232	48	0.800	0.719–0.881
Sodium range	232	48	0.799	0.719–0.880
Intraday sodium range	232	48	0.747	0.666–0.829
Intraday sodium SD	232	48	0.736	0.650–0.822
Interday minimum sodium	232	48	0.729	0.647–0.811
Minimum sodium	232	48	0.701	0.616–0.786
Admission sodium	232	48	0.606	0.509–0.703

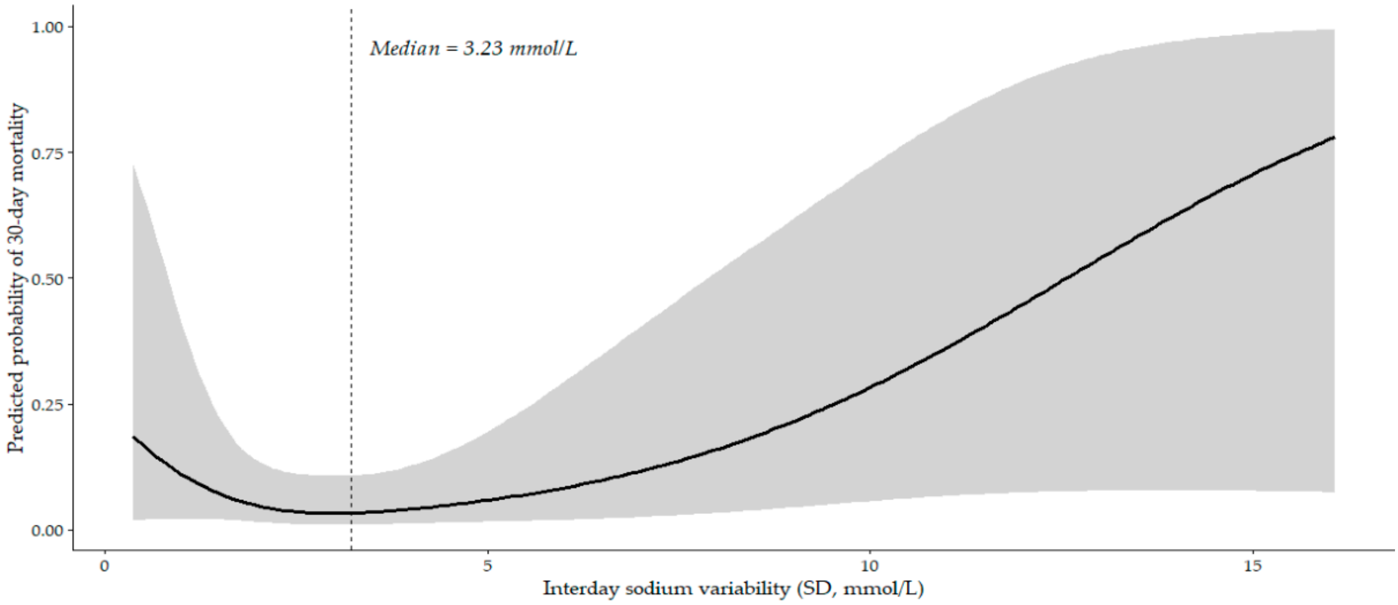

Figure S2. Restricted cubic spline showing the adjusted association between interday sodium variability and 30-day mortality. The model was adjusted for age, sex, WFNS grade, modified Fisher grade, Subarachnoid Hemorrhage Early Brain Edema Score (SEBES), admission glucose concentration, and severe hypernatremia. The solid line represents the predicted probability of 30-day mortality, and the shaded area represents the 95% confidence interval (CI). The vertical

dashed line indicates the cohort median interday sodium variability (3.23 mmol/L). Overall association: P = 0.023; test for nonlinearity: P = 0.125.

Table S3. Pairwise comparison of interday sodium variability with other sodium-derived indices. Pairwise differences in the area under the receiver operating characteristic curve (AUC) were assessed using DeLong's test for paired ROC curves. All comparisons included 232 patients with 30-day mortality as the outcome. ΔAUC represents the difference between the AUC of the comparator index and that of interday sodium SD. P values were adjusted for multiple comparisons using the Benjamini–Hochberg false discovery rate (FDR) procedure. AUC, area under the receiver operating characteristic curve; CV, coefficient of variation; FDR, false discovery rate; ROC, receiver operating characteristic; SD, standard deviation.

Sodium-derived index	AUC	ΔAUC vs interday sodium SD	DeLong *P*	FDR-adjusted *P*
Intraday maximum sodium	0.901	0.086	0.017	0.049
Interday mean sodium	0.894	0.078	0.036	0.087
Interday maximum sodium	0.889	0.073	0.005	0.023
Mean sodium	0.887	0.072	0.057	0.125
Maximum sodium	0.879	0.063	0.014	0.044
Intraday minimum sodium	0.872	0.056	0.189	0.280
Interday sodium delta	0.839	0.024	0.620	0.708
Sodium delta	0.831	0.016	0.756	0.811
Overall sodium SD	0.812	−0.003	0.708	0.777
Mean daily sodium change	0.808	−0.007	0.773	0.820
Interday sodium CV	0.805	−0.010	<0.001	0.004
Interday sodium range	0.804	−0.011	0.308	0.415
Maximum daily sodium change	0.802	−0.014	0.585	0.683
Overall sodium CV	0.800	−0.016	0.074	0.153
Sodium range	0.799	−0.016	0.289	0.394
Intraday sodium range	0.747	−0.069	0.072	0.150
Intraday sodium SD	0.736	−0.079	0.044	0.104
Interday minimum sodium	0.729	−0.087	0.180	0.273
Minimum sodium	0.701	−0.115	0.080	0.160
Admission sodium	0.606	−0.210	0.002	0.011

STROBE Statement—checklist of items that should be included in reports of observational studies

	Item No.	Recommendation	Page No.	Relevant text from manuscript
**Title and abstract**	1	(*a*) Indicate the study's design with a commonly used term in the title or the abstract	1	The abstract identifies the study as a retrospective cohort study.
		(*b*) Provide in the abstract an informative and balanced summary of what was done and what was found	1	Structured abstract including Background, Methods, Results and Conclusions
**Introduction**				
Background/rationale	2	Explain the scientific background and rationale for the investigation being reported	2	The Introduction summarizes current evidence on sodium disturbances after aSAH, identifies knowledge gaps, and explains the rationale for evaluating longitudinal sodium dynamics.
Objectives	3	State specific objectives, including any prespecified hypotheses	2	The study objectives and hypothesis are explicitly stated in the final paragraph of the Introduction.
**Methods**				
Study design	4	Present key elements of study design early in the paper	2–3	Section 2.1 describes the retrospective observational cohort design.
Setting	5	Describe the setting, locations, and relevant dates, including periods of recruitment, exposure, follow-up, and data collection	2–3	Study setting, institution, recruitment period (2014–2025), and clinical environment are described.
Participants	6	(*a*) *Cohort study*—Give the eligibility criteria, and the sources and methods of selection of participants. Describe methods of follow-up<br>*Case-control study*—Give the eligibility criteria, and the sources and methods of case ascertainment and control selection. Give the rationale for the choice of cases and controls<br>*Cross-sectional study*—Give the eligibility criteria, and the sources and methods of selection of participants	2–3	Eligibility criteria, consecutive patient inclusion, exclusion criteria, and final study cohort (n = 232) are reported.
		(*b*) *Cohort study*—For matched studies, give matching criteria and number of exposed and unexposed<br>*Case-control study*—For matched studies, give matching criteria and the number of controls per case	N/A	Not applicable; this was not a matched cohort.
Variables	7	Clearly define all outcomes, exposures, predictors, potential confounders, and effect modifiers. Give diagnostic criteria, if applicable	3	Primary and secondary outcomes, predictors, sodium-derived indices, and diagnostic definitions for DCI and DND are described

Data sources/ measurement	8*	For each variable of interest, give sources of data and details of methods of assessment (measurement). Describe comparability of assessment methods if there is more than one group	3	Clinical data were extracted from the electronic medical record and laboratory database. Sodium-derived indices were calculated from serial measurements.
Bias	9	Describe any efforts to address potential sources of bias	3	Selection bias was minimized through consecutive patient inclusion and predefined eligibility criteria. Confounding was addressed using multivariable regression models adjusted for clinically relevant covariates.
Study size	10	Explain how the study size was arrived at	2–3	The study included all consecutive eligible patients admitted during the predefined study period. No formal sample size calculation was performed because of the retrospective observational design.

Quantitative variables	11	Explain how quantitative variables were handled in the analyses. If applicable, describe which groupings were chosen and why	3–4	Continuous sodium-derived indices (mean sodium, interday SD, intraday SD, sodium range, daily sodium change, etc.) are defined together with outcome measures. The rationale for dichotomization in Kaplan–Meier analyses (median interday sodium variability) is also described.
Statistical methods	12	(*a*) Describe all statistical methods, including those used to control for confounding	4	Student's t-test, Mann–Whitney U test, χ^2 test, Fisher's exact test, Spearman correlation, multivariable logistic and linear regression, Kaplan–Meier analysis with log-rank test, ROC analysis, and linear mixed-effects models were used as appropriate.
		(*b*) Describe any methods used to examine subgroups and interactions	4	Interaction between time and neurological complications (DCI and DND) was evaluated using linear mixed-effects models with interaction terms.
		(*c*) Explain how missing data were addressed	N/A	Patients with incomplete clinical, radiological, or laboratory data were excluded before study inclusion according to predefined eligibility criteria.
		(*d*) *Cohort study*—If applicable, explain how loss to follow-up was addressed<br>*Case-control study*—If applicable, explain how matching of cases and controls was addressed<br>*Cross-sectional study*—If applicable, describe analytical methods taking account of sampling strategy	N/A	Not applicable. This retrospective in-hospital cohort did not involve longitudinal participant follow-up beyond hospital discharge and 30-day mortality assessment from medical records.
		(*e*) Describe any sensitivity analyses	5–10	Sensitivity analyses included restricted cubic spline analyses, alternative multivariable regression models, ROC analyses, and subgroup analyses.
**Results**				

Participants	13 *	(a) Report numbers of individuals at each stage of study—eg numbers potentially eligible, examined for eligibility, confirmed eligible, included in the study, completing follow-up, and analysed	2–3	A total of 323 consecutive patients were screened; after exclusion of 91 patients, 232 were included in the final cohort.
		(b) Give reasons for non-participation at each stage	2–3	Reasons for exclusion are reported with the number of patients in each exclusion category.
		(c) Consider use of a flow diagram	3	A flow diagram was not included because participant selection and reasons for exclusion are comprehensively described in the Methods.
Descriptive data	14 *	(a) Give characteristics of study participants (eg demographic, clinical, social) and information on exposures and potential confounders	5	Baseline demographic, clinical, laboratory, and radiological characteristics are summarized in Table 1.
		(b) Indicate number of participants with missing data for each variable of interest	N/A	Not applicable. Patients with incomplete datasets were excluded before inclusion in the analytical cohort.
		(c) *Cohort study*—Summarise follow-up time (eg, average and total amount)	5	Clinical outcomes were assessed during hospitalization and at 30 days after admission.
Outcome data	15 *	*Cohort study*—Report numbers of outcome events or summary measures over time	5–10	Clinical outcomes, mortality, neurological status, sodium-derived indices, DCI, DND, ROC analyses, Kaplan–Meier analyses, and mixed-effects models are reported.
		Case-control study—Report numbers in each exposure category, or summary measures of exposure		
		Cross-sectional study—Report numbers of outcome events or summary measures		
Main results	16	(*a*) Give unadjusted estimates and, if applicable, confounder-adjusted estimates and their precision (eg, 95% confidence interval). Make clear which confounders were adjusted for and why they were included	6–10	Both unadjusted comparisons and adjusted multivariable regression models are presented with effect estimates and 95% confidence intervals. Adjusted models included age, sex, admission WFNS grade, modified Fisher grade, admission glucose concentration, and, where appropriate, SEBES and severe hypernatremia.
		(*b*) Report category boundaries when continuous variables were categorized	4	Patients were dichotomized according to the cohort median interday sodium variability for Kaplan–Meier analyses.
		(*c*) If relevant, consider translating estimates of relative risk into absolute risk for a meaningful time period	N/A	Absolute risk estimates were not the objective of the present study.

Other analyses	17	Report other analyses done—eg analyses of subgroups and interactions, and sensitivity analyses	7–10	Additional analyses included ROC analyses, Kaplan–Meier survival analyses, restricted cubic spline analyses, interaction analyses, and longitudinal linear mixed-effects modelling.
**Discussion**				
Key results	18	Summarise key results with reference to study objectives	11	The Discussion opens with a concise summary of the principal findings in relation to the study objectives.
Limitations	19	Discuss limitations of the study, taking into account sources of potential bias or imprecision. Discuss both direction and magnitude of any potential bias	15	The retrospective single-center design, observational nature of the study, and possibility of residual confounding are acknowledged.
Interpretati on	20	Give a cautious overall interpretation of results considering objectives, limitations, multiplicity of analyses, results from similar studies, and other relevant evidence	11–15	The findings are interpreted cautiously in the context of previous literature, biological plausibility, and study limitations.
Generalisab ility	21	Discuss the generalisability (external validity) of the study results	15	The need for prospective multicenter studies is discussed, acknowledging the limitations in generalizing the findings.
**Other information**				
Funding	22	Give the source of funding and the role of the funders for the present study and, if applicable, for the original study on which the present article is based	Last page	No external funding

*Give information separately for cases and controls in case-control studies and, if applicable, for exposed and unexposed groups in cohort and cross-sectional studies.

Note: An Explanation and Elaboration article discusses each checklist item and gives methodological background and published examples of transparent reporting. The STROBE checklist is best used in conjunction with this article (freely available on the Web sites of PLoS Medicine at http://www.plosmedicine.org/, Annals of Internal Medicine at http://www.annals.org/, and Epidemiology at http://www.epidem.com/). Information on the STROBE Initiative is available at www.strobe-statement.org.
